# Impact of N-Terminal Tags on De Novo Vimentin Intermediate Filament Assembly

**DOI:** 10.3390/ijms23116349

**Published:** 2022-06-06

**Authors:** Saima Usman, Hebah Aldehlawi, Thuan Khanh Ngoc Nguyen, Muy-Teck Teh, Ahmad Waseem

**Affiliations:** 1 Centre for Oral Immunobiology and Regenerative Medicine, Institute of Dentistry, Barts and The London School of Medicine and Dentistry, Queen Mary University of London, Newark Street, London E1 2AT, UK; s.usman@qmul.ac.uk (S.U.); ntkngoc@web.de (T.K.N.N.); m.t.teh@qmul.ac.uk (M.-T.T.); 2 Department of Oral Diagnostic Sciences, Division of Oral Pathology and Medicine, Faculty of Dentistry, King Abdul Aziz University, Jeddah 21589, Saudi Arabia; haldehlawi@kau.edu.sa; 3 Centre for Immunobiology and Regenerative Medicine, Blizard Institute, 4 Newark Street, London E1 2AT, UK

**Keywords:** intermediate filaments, ectopic protein expression, fusion proteins, immunofluorescence, protein domains

## Abstract

Vimentin, a type III intermediate filament protein, is found in most cells along with microfilaments and microtubules. It has been shown that the head domain folds back to associate with the rod domain and this association is essential for filament assembly. The N-terminally tagged vimentin has been widely used to label the cytoskeleton in live cell imaging. Although there is previous evidence that EGFP tagged vimentin fails to form filaments but is able to integrate into a pre-existing network, no study has systematically investigated or established a molecular basis for this observation. To determine whether a tag would affect de novo filament assembly, we used vimentin fused at the N-terminus with two different sized tags, AcGFP (239 residues, 27 kDa) and 3 × FLAG (22 residues; 2.4 kDa) to assemble into filaments in two vimentin-deficient epithelial cells, MCF-7 and A431. We showed that regardless of tag size, N-terminally tagged vimentin aggregated into globules with a significant proportion co-aligning with β-catenin at cell–cell junctions. However, the tagged vimentin aggregates could form filaments upon adding untagged vimentin at a ratio of 1:1 or when introduced into cells containing pre-existing filaments. The resultant filament network containing a mixture of tagged and untagged vimentin was less stable compared to that formed by only untagged vimentin. The data suggest that placing a tag at the N-terminus may create steric hinderance in case of a large tag (AcGFP) or electrostatic repulsion in case of highly charged tag (3 × FLAG) perhaps inducing a conformational change, which deleteriously affects the association between head and rod domains. Taken together our results shows that a free N-terminus is essential for filament assembly as N-terminally tagged vimentin is not only incapable of forming filaments, but it also destabilises when integrated into a pre-existing network.

## 1. Introduction

Vimentin is a type III intermediate filament (IF) [1] protein with 466 residues and a molecular mass of 53.7 kDa which is normally expressed in mesenchymal tissues and in cells derived from them. It is well organized in the cytoplasm and is also found on the nuclear and cellular surfaces [2]. Vimentin IFs closely coordinate with other cytoskeleton components to act as a major modulator of cellular dynamics especially migration, cellular attachment, molecular trafficking, signalling and transcriptional regulation [3]. It is also responsible for subcellular localization of other organelles, especially nucleus and mitochondria [4,5]. One of the important functions of vimentin is to help the cells adapt to various kinds of stresses including heat [6,7,8], oxidative and electrophilic addition [9]. It is considered a marker of epithelial mesenchymal transition (EMT) and is highly expressed during cancer metastasis [10,11]. Vimentin can also influence extracellular matrix (ECM) metabolism as it binds and stabilises collagen mRNA [12].

Each vimentin monomer contains a highly conserved central α-helical rod domain of 312 residues, flanked by non-helical head (N-terminal) and tail (C-terminal) domains. The rod domain is further split into 1A, 1B (together forming coil 1), 2A and 2B (together forming coil 2) α-helical segments that are interspaced by L1, L12 and L2 linkers [13,14,15], although recent literature on the subject has challenged the existence of linker L2 [16]. Towards the C-terminus of coil 2 at position 350 there is a highly conserved region called ‘stutter’ causing discontinuity in the heptad repeats [17]. The central domain contains heptad (**a**-b-c-**d**-e-f-g) repeats which form an α-helical structure. The first and the fourth residues (‘a’ and ‘d’) in the heptad are always leucine or isoleucine, which provides a hydrophobic patch on the helix surface giving a suitable assembly environment for the two monomers to associate in register to form a coiled-coil dimer [18,19]. Further association of dimers in an antiparallel and staggered fashion in three different alignments produces tetramers [15,20,21], and eight tetramers laterally assemble to form 60 nm long unit length filaments (ULFs) [22]. The ULFs then undergo further association in head to tail fashion to produce 17nm thick filaments which is followed by ionic strength dependent radial compaction to produce 10nm mature IFs [23,24,25]. The filamentous vimentin network interacts with cytoplasmic proteins including actin [26,27,28], dynactin, dynein and kinesin [29,30].

The exact role of head, rod and tail domains of vimentin in filament assembly is still inexplicit. Studies using bacterially expressed recombinant vimentin have shown that head and rod domains are crucial for positioning of both end-to-end and lateral associations during vimentin assembly in vitro [13]. Detailed analysis using deletion mutagenesis showed that the middle region of the head domain loops out in filaments and may become accessible to other cytoplasmic proteins [31]. These studies have also shown an association between the head and rod domains necessary for filament assembly [31,32]. Using site-directed spin labelling (SDSL) and electron paramagnetic resonance (EPR), FitzGerald and his group showed that the head domain folds back and interacts with the rod domain, which is essential for filament formation and stabilization [33]. The head and the tail domains are also able to keep the vimentin network extended between the nucleus and cell periphery [34]. The tail domain plays a role in maintaining the filament diameter as well as regulating the speed of filament assembly [13]. The head domain is reported as the major site for posttranslational modifications including phosphorylation, O-glycosylation, ADP ribosylation, proteolysis by Ca^++^ activated neutral thiol proteinases and deamination of arginine residues that are instrumental in assembly and disassembly of IF [35].

Several approaches have been used to study the role(s) of different vimentin domains in in vivo filament assembly. The most common is the truncation of the different vimentin domains and studying their effects on filament assembly by tagging them to specific epitopes such as FLAG (or 3 × FLAG), Myc and fluorescent proteins [36]. Fluorescent tags can be easily visualised under microscope and can also be used in live cell imaging [37]. Other approaches include dominant negative constructs to disrupt the preformed assembly, biotinylated vimentin, rhodamine labelled vimentin, siRNA, synthetic peptides and drug molecules such as withaferin A, diamide, Taxol, etc. [38].

It has been previously reported that EGFP tagged vimentin is unable to form filaments in most of SW13 Vim− but formed normal filaments in some SW13 Vim− cells. It was also shown that EGFP-vimentin did not form filaments in vimentin deficient murine MFT16 cells but was able to integrate into a pre-existing network [39,40]. However, studies have not compared the effect of different commonly used tags and the possible mechanistic reasons for the failure of filament assembly. Here we report the inability of the full-length vimentin fused with two different size tags (AcGFP vs. 3 × FLAG) at the N-terminus to assemble into filaments in two vimentin deficient epithelial cells, MCF-7 and A431. We have not evaluated the filament forming ability of the C-terminally tagged vimentin because the C-terminus is known to interact with actin cytoskeleton [26,27,28] and does not appear to participate in the filament formation [41]. Thus, placing a tag at the C-terminus of vimentin is predicted to have very little or no impact on filament assembly. On the other hand, a tag at the N-terminus is likely to be interesting as the head domain is reported to fold back and interact with the rod domain [33,42]. However, the interaction between head and rod has so far been shown only biochemically and there is no in vivo data to support their role in filament assembly. Our results show that the N-terminally tagged vimentin only forms cytoplasmic globules, and a significant proportion of them accumulate at the intercellular junctions, where they co-align with β-catenin. We also show that the N-terminally tagged vimentin can form filaments upon adding untagged vimentin or when introduced into cells containing pre-existing filaments, although the presence of tagged vimentin appears to weaken the network. Our data are consistent with the hypothesis that a large tag or a charged tag at the N-terminus prevents filament assembly, perhaps by disrupting head–head and head–rod interactions.

## 2. Results

### 2.1. Filament Formation by Ectopically Expressed Untagged Vimentin (UTV) in the Absence of Endogenous Vimentin

**Figure 1:**
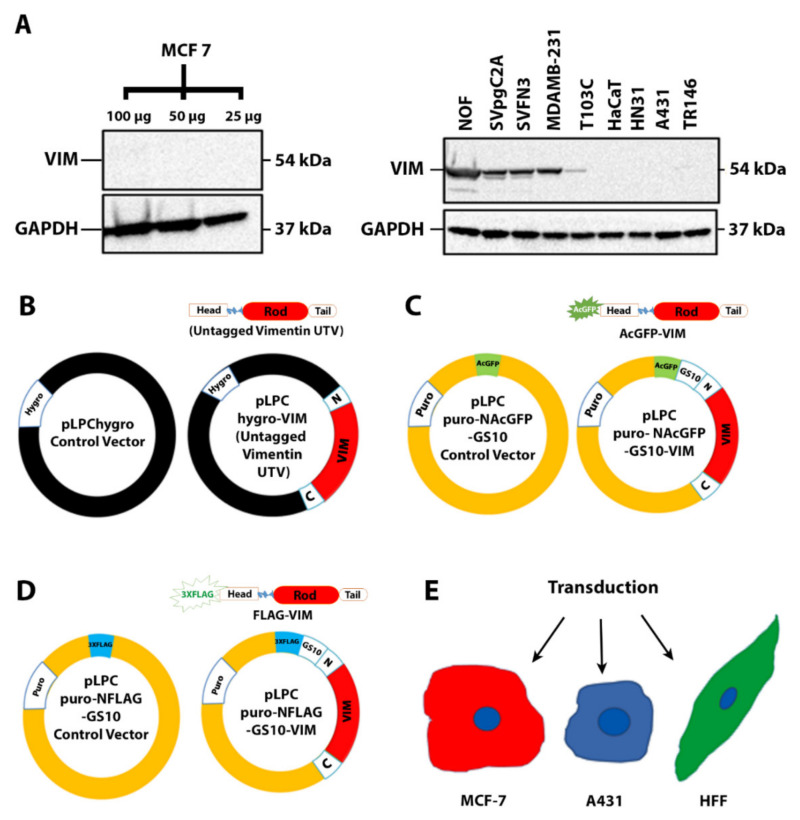
**Graphical representation of different constructs used in this study**. (**A**) Vimentin expression analysed in 10 different cancer cell lines by western blotting. A total of 50 µg total protein was loaded for all other cell lines; for MCF-7, 100, 50 and 25 µg protein were loaded onto SDS PAGE gels. GAPDH was used as the loading control. Relevant bands were cropped from different gels and regrouped. Original blots are shown in Supplementary Figure S1. (**B**) Construct pLPChygro-VIM expressing untagged vimentin (UTV) and pLPChygro its control vector (CV); N and C are the 5′ and 3′ ends of vimentin cDNA, respectively, black color represents the vector backbone. (**C**) pLPCpuro-NAcGFP-GS10-VIM expressing AcGFP-VIM and pLPCpuro-NAcGFP-GS10 expressing AcGFP its CV. (**D**) pLPCpuro-NFLAG-GS10-VIM expressing FLAG-VIM and pLPCpuro-NFLAG-GS10 expressing FLAG its CV, the FLAG tag was 3 × FLAG, yellow colour in B and C represents vector backbone. (**E**): MCF-7, A431 and HFF-1 cell lines (nuclei in blue and the cytoplasm is shown in different colors) were transduced with the constructs described in (**B**–**D**).

**Figure 2:**
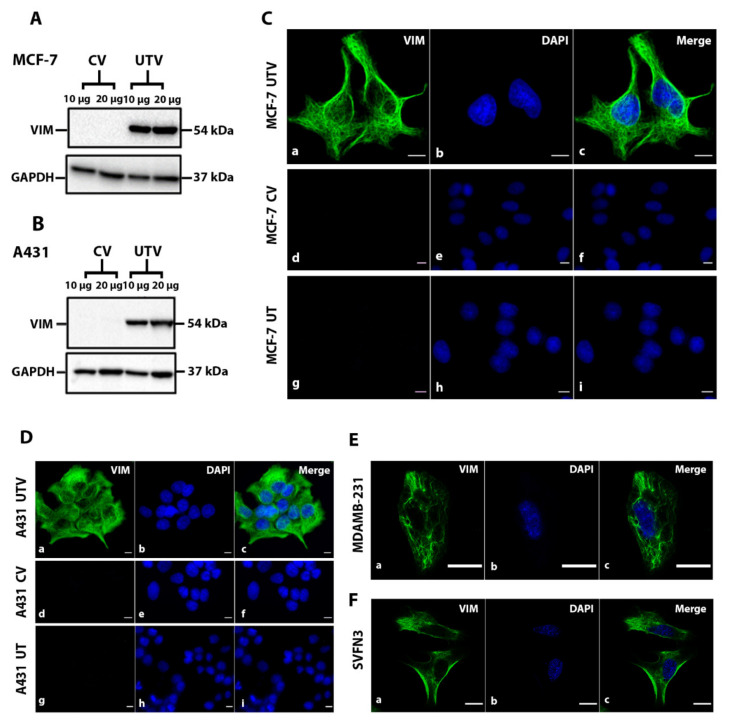
**Ectopic expression of vimentin in vimentin-deficient cells.** (**A**) MCF-7 and (**B**) A431 cells were transduced with the untagged vimentin (UTV) and its control vector (CV) by recombinant retrovirus supernatant. Total protein extract, 10 and 20 µg, from each transduced cell line were loaded onto SDS PAGE gels, transferred onto nitrocellulose and probed with anti-vimentin antibody to confirm the transduction efficiency. GAPDH was used as the loading control. Relevant bands were cropped from different gels and regrouped. Original blots are shown in the Supplementary Figure S2. Immunostaining of (**C**) MCF-7 and (**D**) A431 cells transduced with CV and UTV constructs. Untransduced (UT) MCF-7 and A431 cells were also used as controls. Cells were immunostained with anti-vimentin antibody and counter stained with AF-488-labelled goat anti-mouse secondary antibody showing green fluorescence. Nuclei were stained with DAPI in blue and overlapping images are shown as ‘Merge’. Leica DM4000B Epi-fluorescence microscope and DFC350 camera were used for recording images. Immunostaining of (**E**) MDA-MB-231 and (**F**) SVFN3 cells expressing endogenous vimentin were also immunostained with anti-vimentin antibody. The vimentin staining in SVFN3 and MDA-MB-231 cells was very similar to that in transduced MCF-7 and A431cells shown in C and D (scale bar = 20 µm).

To identify the vimentin-deficient cells, we analysed 10 different epithelial cell lines (Table 1) for the expression of endogenous vimentin by western blotting. Among these, endogenous vimentin was undetectable in HaCaT, MCF-7, HN31 and A431, whereas a very faint band was barely visible in TR146 (Figure 1A). Based on this analysis, we chose two epithelial cell lines, MCF-7, a simple epithelial cell line, and A431, a vulval keratinocyte cell line for further studies. We made a series of untagged and tagged vimentin constructs (Figure 1B–D), described in Methods and Materials, to analyse their expression in MCF-7 and A431 (Figure 1E). We transduced both cell lines with recombinant retrovirus expressing either the control vector (CV; pLPChygro) or the untagged vimentin (UTV; pLPChygro-VIM). The western blot analysis of the stable clones confirmed that untagged vimentin was expressed in both MCF-7 (Figure 2A) and A431 cells (Figure 2B). The MCF-7 (Figure 2C) and A431 (Figure 2D) clones stably expressing untagged vimentin on immunostaining with anti-vimentin antibody showed filament formation in both cell lines but not in cells expressing the control vector. To demonstrate that the untagged vimentin filaments in MCF-7 and A431 cells appeared similar to those endogenously expressed, we immunostained two epithelial cell lines, MDA-MB-231 cells and SVFN3, endogenously expressing vimentin. As shown in Figure 2E (MDA-MB-231) and Figure 2F (SVFN3), the naturally occurring filaments were very similar to those formed by untagged vimentin in MCF-7 (Figure 2C) and A431 (Figure 2D) cells.

### 2.2. Ectopic Expression of N-Terminally Tagged Vimentin in Cells Devoid of Endogenous Vimentin

**Figure 3:**
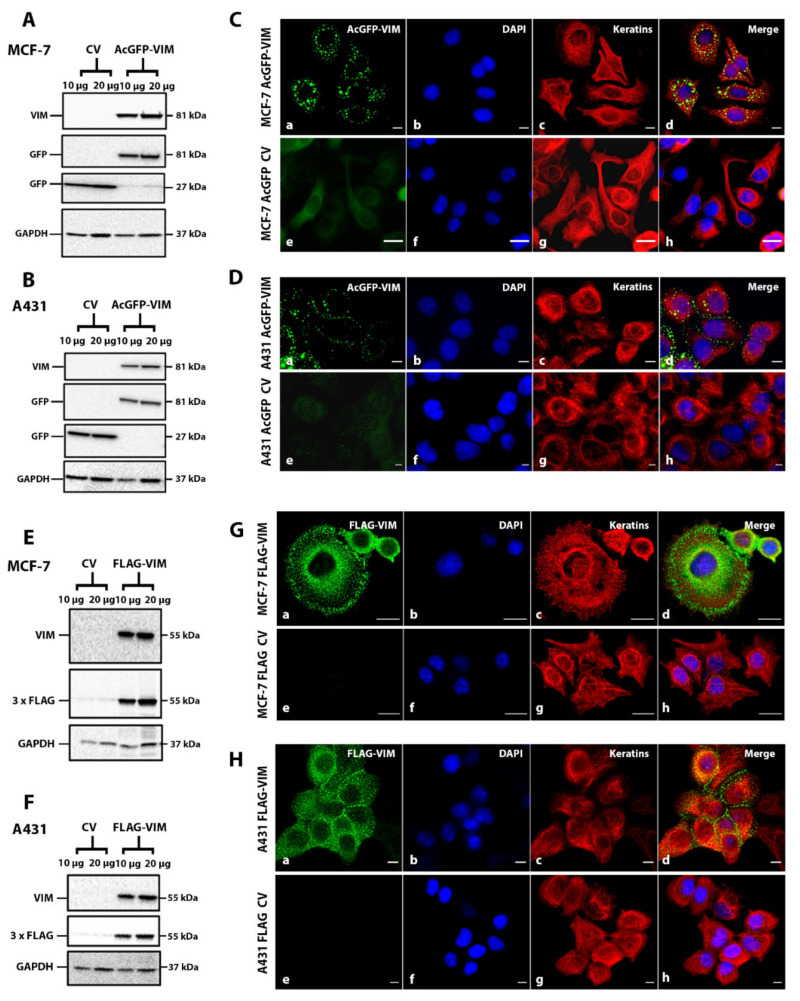
**Ectopic expression of N-terminally tagged vimentin in MCF-7 and A431 cells.** Healthy growing cells (**A**,**E**) MCF-7 and (**B**,**F**) A431 were transduced separately with (**A**,**B**) control vector (CV) AcGFP and AcGFP-VIM; (**E**,**F**) control vector (CV) FLAG and FLAG-VIM, respectively. Total protein extract, 10 and 20 µg, from each transduced cell line was loaded onto SDS PAGE gels, transferred onto nitrocellulose, and probed with mouse anti-vimentin antibody, or rabbit anti-FLAG antibody to confirm the transduction efficiency. GAPDH was used as the loading control. Molecular weights of the gel bands are given on the right-hand side of the blots. Relevant bands were cropped from different blots and regrouped together. Original blots are shown in Supplementary Figures S3 and S4. (**C**,**G**) MCF-7 and (**D**,**H**) A431 cells expressing (**C**,**D**) AcGFP and AcGFP-VIM and (**G**,**H**) FLAG and FLAG-VIM, respectively, were grown on collagen coated glass coverslips and fixed with acetone: methanol (1:1). The cells in (**G**,**H**) were incubated with rabbit anti-FLAG antibody and counterstained with AF-488 goat anti-rabbit secondary antibody (green). All coverslips in (**C**,**D**,**G**,**H**) were incubated with A45-B/B3 (keratins) and counter stained with AF-594 labelled goat anti-mouse secondary antibody (red). Nuclei were stained with DAPI in blue and overlapping of all colours is shown as ‘Merge’. Leica DM4000B Epi-fluorescence microscope was used for visualisation and DFC350 camera was used for image recording (scale bar = 20 µm).

To investigate whether the N-terminally tagged vimentin would form de novo IFs, we transduced MCF-7 and A431 cells with recombinant retroviruses expressing either AcGFP or AcGFP-VIM. Western blotting data showed that in both MCF-7 (Figure 3A) and A431 cells (Figure 3B), the vimentin and GFP antibodies independently recognised a single band of about 81 kDa suggesting the in-frame fusion of AcGFP (27 kDa) with vimentin (54 kDa). The control vector cells expressing AcGFP alone had only one 27 kDa band reacting with anti-GFP antibody (GFP antibodies cross react with AcGFP). Visualisation of the cells expressing AcGFP-VIM showed that the N-terminally tagged vimentin failed to form filaments in both MCF-7 (Figure 3C) and in A431 (Figure 3D) cells, instead aggregated into globules in the cytoplasm. These globules were of different sizes and a significant proportion were arranged around the cell periphery especially in cells forming a colony. No observable organisational differences were found in keratin filaments of both cell types expressing either AcGFP or AcGFP-VIM (Figure 3C–H (compare panels c with g).

We hypothesised that the large size of AcGFP may have interfered with intermolecular association thereby preventing the formation of vimentin filament. To test this hypothesis, we substituted AcGFP with 3 × FLAG, which would reduce the tag size from 239 to 22 residues, to investigate if a smaller tag size could avoid formation of vimentin globules. Western blotting analysis of the FLAG-VIM transduced MCF-7 (Figure 3E) and A431 (Figure 3F) cells showed that a single vimentin band reactive with anti-vimentin and anti-FLAG antibodies confirmed that the oligo for 3 × FLAG was fused in frame with vimentin cDNA. Double immunofluorescence analysis of these cells with anti-FLAG antibodies showed that FLAG-VIM also did not form filaments, but instead aggregated into globules of similar sizes in MCF-7 (Figure 3G) and A431 cells (Figure 3H). These globules showed similar distribution as those seen in cells expressing AcGFP-VIM (Figure 3C,D), with most of them organised at the cell periphery (Figure 3H(a–d)). In addition to globule formation, the FLAG-VIM also gave a hazy appearance around the nucleus in both cell types which was not observed with AcGFP-VIM (compare Figure 3C,D with Figure 3G,H). The control vector of both AcGFP and 3 × FLAG in both cell types gave only diffuse staining in the cytoplasm (Figure 3C,D,G,H, in e–h panels). Collectively, the data presented in Figure 3 suggested that tagging vimentin at the N-terminus interfered with filament assembly and makes vimentin incapable of forming filaments irrespective of the tag size used.

### 2.3. Co-Alignment of N-Terminally Tagged Vimentin Aggregates with β-Catenin

**Figure 4:**
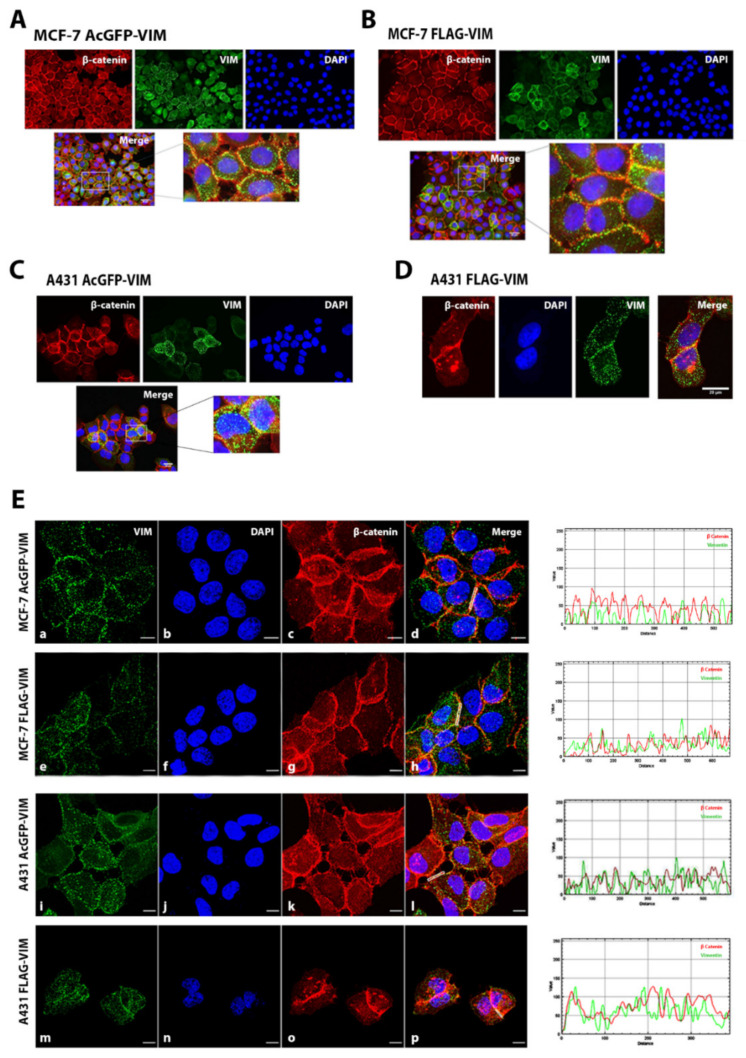
**Co-alignment of vimentin globules with β-catenin in MCF-7 and A431 cells at intercellular junction.** (**A**,**B**) MCF-7 and (**C**,**D**) A431 cells stably expressing (**A**,**C**) AcGFP-VIM and (**B**,**D**) FLAG-VIM were grown on collagen coated glass coverslips. The cells were immunostained with mouse anti-β catenin antibody and AF-594-labelled goat anti-mouse as secondary antibody (red). For cells expressing 3 × FLAG tagged vimentin, cells were stained with rabbit anti-FLAG antibody and AF-488 labelled goat anti-rabbit secondary antibody (green). Nuclei were stained with DAPI in blue and all overlapping images are shown as ‘Merge’. Leica DM4000B Epi-fluorescence microscope was used for visualisation and DFC350 camera was used for recording. Marked areas showing intercellular junctions were magnified for clarity in panels (**A**–**C**). (**E**) Images taken by Zeiss 880 laser scanning confocal microscope with Fast Airyscan and Multiphoton (inverted) system. Co-alignment between vimentin aggregates and β catenin is shown by line graph drawn using RGB profiler on maximal single confocal sections. The area used for this analysis is shown by a white line in different panels (scale bar = 20 µm).

As we observed that N-terminally tagged vimentin aggregates (for both AcGFP and FLAG) were organised mostly at the cell periphery in A431 and MCF-7 cells (Figure 3C–H), we further investigated whether these aggregates were co-localising with any of the intercellular junctional protein. Cells were double immunostained with anti-VIM or anti-FLAG and antibodies specific to E-cadherin, vinculin, α6 integrin and β-catenin. The co-alignment of vimentin with β-catenin is shown in Figure 4A–D. The analysis of data using the RGB profiler tool in *image J* software developed at the National Institute of Health, USA (freely available at https://imagej.nih.gov/ij/download.html bundled with 64‐bit Java1.8.0_112 (accessed on 4 September 2020)) showed co-alignment of vimentin aggregates with only β-catenin in both MCF-7 and A431 cells (Figure 4E) and not with E-cadherin, vinculin or α6 integrin (Supplementary Figure S6). The quantification of the data using Coloc plugin in *image J* software in case of β-catenin however showed no colocalization. Similarly, our β-catenin was not co-immunoprecipitated with vimentin (Supplementary Figure S7).

### 2.4. N-Terminally Tagged Vimentin Did Not Act as Dominant Negative

**Figure 5:**
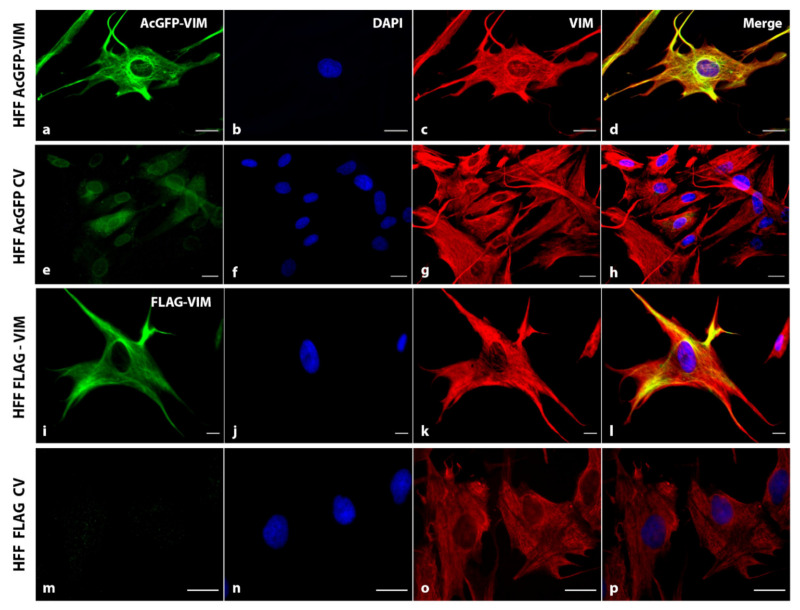
**Integration of N-terminally tagged vimentin into the pre-existing vimentin filaments**. HFF-1 cells expressing AcGFP-VIM, control vector (CV) AcGFP, FLAG-VIM and control vector (CV) FLAG were immunostained with anti-VIM antibody (for endogenous vimentin network) and AF-594-labelled goat anti-mouse as secondary antibody (red). For cells expressing 3 × FLAG constructs, cells were stained with anti-FLAG antibody and AF-488 labelled goat anti-rabbit as secondary antibody (green). Nuclei were stained with DAPI in blue and all overlapping images are shown as ‘Merge’. Leica DM4000B Epi-fluorescence microscope was used for visualisation and DFC350 camera was used for image recording (scale bar = 20 µm). Note that N-terminally tagged vimentin (green colour panels (**a**,**i**)) has integrated into the endogenous vimentin (red colour panel (**c**,**k**)) filament network in HFF-1 cells which becomes yellow (**d**,**l**) in ‘Merge’. No disruption of pre-existing vimentin network was seen in these cells. The western blot analysis showed that the transduced HFF-1 cells had tagged and endogenous vimentin in a ratio of 1:1 (Figure S5).

To investigate whether tagging vimentin at the N-terminus renders dominant negative behaviour, we transduced the AcGFP-VIM and FLAG-VIM into human foreskin fibroblast (HFF-1) cell line containing pre-existing endogenous vimentin filaments. We hypothesised that if the N-terminally tagged vimentin was acting as dominant negative, then adding it into HFF-1 will disrupt the pre-exiting filaments. As shown in Figure 5, transduction of vimentin tagged with either AcGFP (Figure 5a–d) or 3 × FLAG (Figure 5i–l) into HFF-1 did not cause aggregation of pre-existing filaments; instead, it did assemble into filaments. The AcGFP fluorescence (Figure 5a–d) and anti-FLAG immunostaining ((Figure 5i–l) co-localised with immunostaining using the anti-vimentin antibody, suggesting that the N-terminally tagged vimentin had in fact integrated into the pre-existing filaments. Detection of AcGFP-vimentin and endogenous vimentin using anti-vimentin antibody has the limitation that the antibody will not specifically detect the endogenous vimentin network; instead, it will bind to both AcGFP-tagged and endogenous vimentin network. The protein analysis by western blot (Figure S5) showed a 1:1 ratio between AcGFP-tagged and endogenous vimentin in HFF-1 cells.

### 2.5. Aggregation of N-Terminally Tagged Vimentin Was Reversible

**Figure 6:**
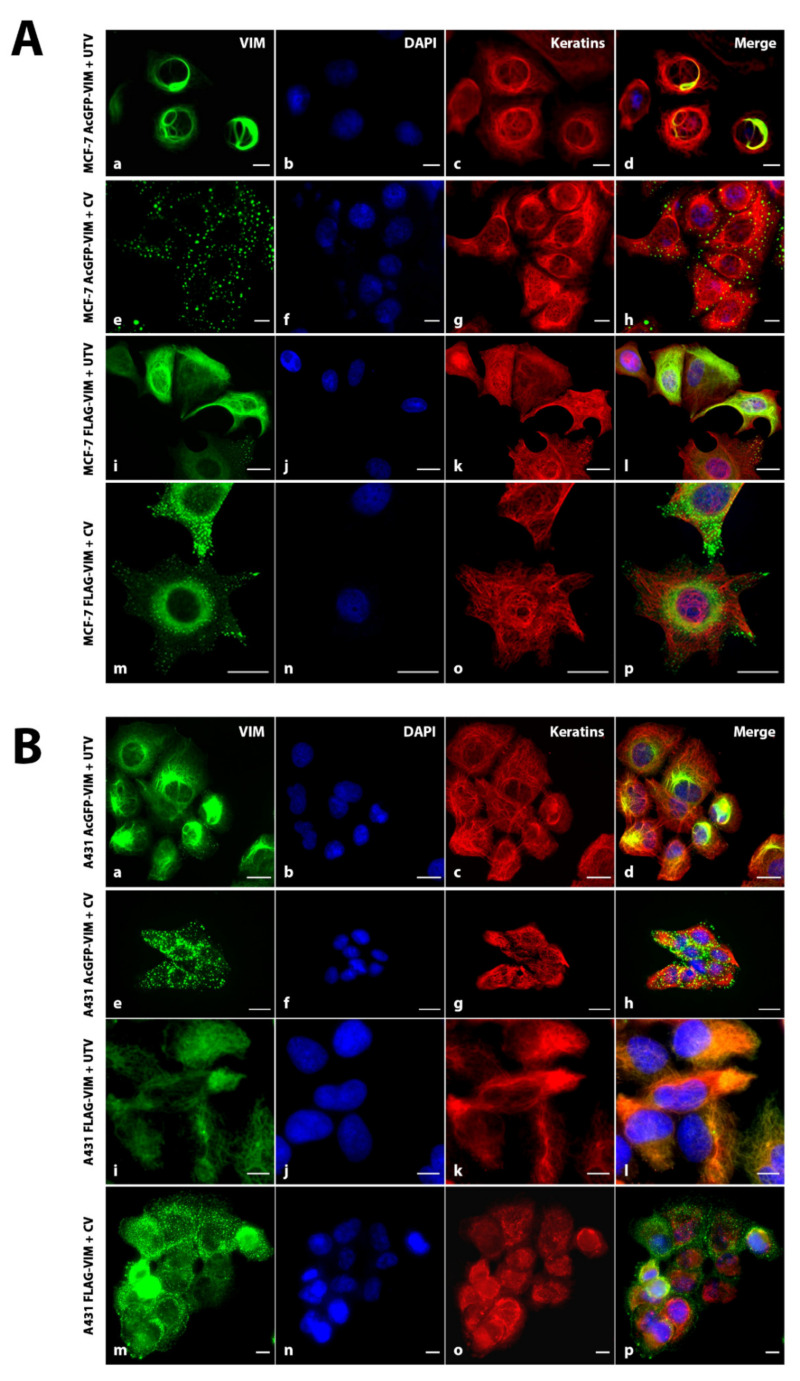
**Aggregates of N-terminally tagged vimentin can be reversed by untagged vimentin (UTV).** (**A**) MCF-7 and (**B**) A431 cells expressing AcGFP-VIM and FLAG-VIM vimentin aggregates were transduced with untagged vimentin (UTV) virus. The control vector (CV) expressed either AcGFP or 3 × FLAG. After 48 h the cells were fixed in acetone:methanol (1:1) and stained with A45-B/B3 for keratins and AF-594-labelled goat anti-mouse secondary antibody (red). For cells expressing FLAG-VIM, the cells were stained with anti-FLAG antibody and AF-488-labelled goat anti-rabbit secondary antibody (green). Nuclei were stained with DAPI in blue and overlapping of all colours is shown as ‘Merge’. Leica DM4000B Epi-fluorescence microscope was used for visualisation and DFC350 camera was used for image recording. (scale bar = 20 µm). Note that vimentin aggregation has been completely reversed by UTV and filaments can be seen in green colour (**a**,**i**). The western blot analysis shows a 1:1 ratio between AcGFP-tagged and untagged vimentin (Figure S5).

To further investigate whether the cytoplasmic globules formed from AcGFP-VIM and FLAG-VIM can form filaments in the presence of untagged vimentin, we transduced MCF-7 and A431, stably expressing AcGFP-VIM or FLAG-VIM with untagged vimentin or the control vector. We observed that the N-terminally tagged vimentin globules of AcGFP-VIM and FLAG-VIM were able to form filaments in both MCF-7 (Figure 6A) and A431 (Figure 6B) cells. Western blot analysis was carried out to determine the ratio between the tagged and untagged vimentin in MCF-7 and A431 cells transduced with AcGFP-VIM or FLAG-VIM with untagged vimentin, UTV. The data shows a 1:1 ratio between AcGFP-tagged and untagged vimentin. 3 × FLAG tagged and untagged vimentin could not be separated on SDS PAGE as the difference between their molecular weights (2.4 kDa) was not high enough (Figure S5).

### 2.6. Response of Ectopically Expressed Vimentin Filament Network to Oxidative Stress

**Figure 7:**
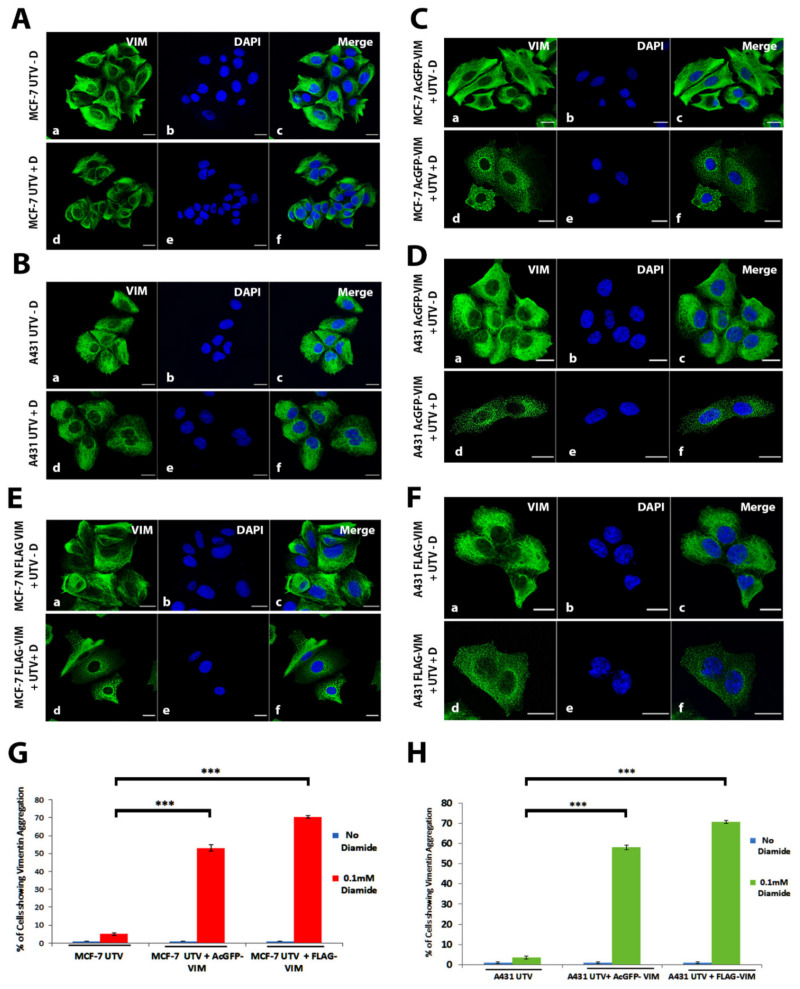
**Destabilisation of vimentin network by integration of tagged vimentin.** (**A**) MCF-7 and (**B**) A431 cells expressing untagged vimentin (UTV) were treated with (+D) or without (−D) 0.1 mM diamide for 10 min and immunostained with anti-vimentin antibody; (**C**,**E**) MCF-7 and (**D**,**F**) A431 cells expressing (**C**,**D**) AcGFP-VIM and (**E**,**F**) FLAG-VIM, respectively, were transduced with UTV and treated with (+D) or without (−D) 0.1 mM diamide for 10 min. The AcGFP expressing cells were visualised after mounting whereas the FLAG expressing cells were immunostained with anti-FLAG antibody. Quantification of cells with disrupted vimentin network as a result of diamide treatment in (**G**) MCF-7 and (**H**) A431 cells. About 200 cells were visually evaluated for vimentin aggregation under Epi-fluorescence microscope in triplicates for every set. Collapse of filaments by diamide was observed only in 6 ± 0.72% MCF-7 and 3.6 ± 0.91% A431 cells expressing untagged vimentin UTV alone. In UTV + AcGFP-VIM expressing cells, diamide caused filament collapse in 53 ± 1.69% (*** *p* < 0.001) MCF-7 and 51 ± 0.24% (*** *p* < 0.001) A431 cells. In UTV + FLAG-VIM expressing cells, diamide caused vimentin aggregation in 60 ± 0.72% (*** *p* < 0.001) MCF-7 and 63 ± 0.92% (*** *p* < 0.001) A431 cells, the p values indicate the data are extremely significant. (scale bar = 20 µm).

Having established that N-terminally tagged vimentin alone cannot form filaments, but they can incorporate themselves into filamentous network when untagged vimentin is introduced in MCF-7 or A431 cells, led us to hypothesise that incorporation of N-terminally tagged vimentin into untagged vimentin network may produce weaker filaments. To test this hypothesis, we exposed MCF-7 and A431 cells expressing untagged vimentin and AcGFP-VIM or FLAG-VIM with or without diamide (0.1 mM for 10 min) to induce oxidation and destabilisation of filaments [9,43]. The integrity of vimentin network was evaluated by fluorescence microscopy after immunostaining. The results showed that exposure of diamide caused collapse of vimentin network into aggregates (Figure 7A–F). The collapse was observed in only 6 ± 0.72% and 3.6 ± 0.91% of diamide treated MCF-7 and A431 cells expressing untagged vimentin (UTV) alone, respectively (Figure 7G,H). However, diamide caused significant, 53 ± 1.69% (*p* < 0.001) and 60 ± 0.72 (*p* < 0.001) vimentin aggregation in UTV + AcGFP-VIM and UTV + FLAG-VIM in MCF-7, respectively, 51 ± 1.24 (*p* < 0.001) and 63 ± 0.92% (*p* < 0.001) vimentin aggregation in UTV + AcGFP-VIM and UTV + FLAG-VIM in A431 cells, respectively (Figure 7G,H). Our observations suggested that integration of N-terminally tagged vimentin into pre-existing vimentin network, reduces the stability, and weakens the network irrespective of the size of the tag.

## 3. Discussion

Vimentin and other cytoskeletal proteins tagged at the N- or C-terminus have been widely used to study protein dynamics [38]. Most tags are relatively large fluorescent proteins such as EGFP (239 residues, 26.9 kDa), AcGFP (239 residues; 26.9 kDa), Des-Red (238 residues, 27.2 kDa) and m-Cherry (236 residues, 26.7 kDa), which are fused in frame with the protein of interest [4,44,45,46,47]. These fusion proteins have been used in combination with untagged vimentin or transfected into vimentin expressing cells in order to study in vivo filament dynamics in live cells [40], fluorescence recovery after photobleaching [43] and fluorescence energy transfer to study intermolecular interactions [48]. Apart from these fluorescent tags, there are non-fluorescent tags such as N-Myc (EQKLISEEDL) [49,50], FLAG (DYKDDDDK) and 3 × FLAG [51,52], HA Tag (YPYDVPDYA) [53], Spot Tag (PDRVRAVSHWSS) [54,55], V5 Tag (GKPIPNPLLGLDST) [56] and His Tag (HHHHHH) [57,58]. These are small non-fluorescent peptides, which are fused in frame with the protein of interest so the fusion proteins can be tracked using tag-specific antibodies and therefore they are not suitable for live cell imaging.

In this study we show that AcGFP fused at the N-terminus of vimentin failed to form filaments in MCF-7 and A431 cells, instead aggregated into globules spreading in the cytoplasm. This suggests that aggregation of N-terminally tagged vimentin is independent of cell type or cytoskeletal features such as keratin polypeptide composition which is different between MCF-7 and A431 cells. Although this observation is consistent with other studies where similar aggregation of N-terminal tagged vimentin has been observed [39,40], however the data are not very consistent. For example, Ho and co-workers observed formation of aggregates of N-terminally tagged vimentin in SW13VIM^—^ cells but occasionally observed a ‘filamentous pattern’, perhaps due to ‘low degree of revertant’. They also observed formation of a mixture of aggregates and short filaments in 50% of a vimentin knockout murine MFT16 cells from the N-terminally tagged vimentin [39]. Yoon and co-workers also observed a ‘typical filament’ formation in only 1% of SW13VIM^—^ cells transfected with GFP-vimentin, and they explained their observation on “leaky expression” [40]. Clearly SW13VIM^—^ are either not completely vimentin deficient or they are prone to undergo significant reversion. In our hands, however, we did not observe any filamentous pattern of N-terminally tagged vimentin in MCF-7 and A431 despite growing these cells in culture for several weeks at a time over a period of several years.

We have shown that aggregates formed by N-terminally tagged vimentin were of various sizes in both cell lines and mostly distributed at the cell periphery in confluent cells. Using double immunocytochemistry employing antibodies against several intercellular proteins, we observed that vimentin aggregates co-align only with β-catenin and not with E-cadherin, vinculin or α6 integrin, at the intercellular junctions. However, there was no co-immunoprecipitation of β-catenin with AcGFP or FLAG-VIM in pull down assays (Figure S7) suggesting that the association between β-catenin and vimentin (direct or indirect) may not be stable. β-catenin is an important member of intercellular E-cadherin junctional complexes that maintains cell to cell adhesion [59]. During cancer metastasis it is extensively accumulated in the cytoplasm and transported to the nucleus to act as co-activator of TCF/LEF-1 signalling pathway [60]. Phosphorylated vimentin is reported to be an important controlling factor in maintaining cellular junctions, but a co-alignment of vimentin and β-catenin at the intercellular junction has never been reported [61].

Contrary to the association of N-terminally tagged vimentin at the intercellular junctions in MCF-7 and A431 cells, the untagged vimentin only formed cytoplasmic filaments with no obvious localisation to the intercellular junctions, which is consistent with previous studies [62,63]. It can be argued that the conformational alterations caused by placing a tag at the N-terminus exposes a cryptic site which mediates its association to the intercellular junctions. An alternative explanation, however, could be that the affinity between vimentin polypeptides and intercellular junctional complex is low compared with the inter-polypeptide associations in vimentin network, so when untagged vimentin is introduced in the cell they assemble into filament and do not attach with cell–cell junctions due to low affinity. However, in the absence of untagged vimentin, the N-terminally tagged vimentin can only forms globules, which then get associated with the low affinity sites at the intercellular junctions. This is supported by the fact that the globules of N-terminally tagged vimentin in MCF-7 and A431 cells disappear from the intercellular junctions when untagged vimentin are introduced into these cells (Figure 6A,B). It is tempting to speculate that the untagged vimentin in the presence of N-terminally tagged vimentin globules also associates into filaments, with much higher affinity between vimentin polypeptides, so the N-terminally tagged vimentin is pulled away from the low affinity sites at the cell–cell junctions and integrated into the high affinity network formed by the untagged vimentin. This is supported by our observation that addition of UTV can convert most of the globules into filaments, but occasionally tiny dots remain at cell–cell junction (Figure S8).

We also show that the N-terminally tagged vimentin was able to integrate into the pre-existing endogenous vimentin filaments of HFF-1 cells (Figure 5) suggesting that the tagged vimentin did not act as dominant negative as has been reported for Vim_1-138_ [44] and VimR113C [64]. Similar observations have been reported by other workers [39,40,65,66]. It is interesting to note that vimentin staining in these experiments appears to co-localise with keratin staining (green and red turning yellow when they are merged) (Figure 6A(d,l),B(d,l)), which is consistent with the data reported previously [67]. Treatment of vimentin network with diamide (an oxidant) is also reported to disrupt filament network into globules [9,43], in a reversible fashion (Usman et al.; unpublished observations). However, the diamide-induced vimentin globules were different in their cytoplasmic distribution from those produced by placing a tag at the N-terminus. The globules generated by diamide did not align to intercellular junctions unlike those generated by placing a tag at the N-terminus of vimentin (compare Figure 4 and Figure 7). The diamide induced filament disruption was seen significantly higher in filaments formed from a mixture of N-terminally tagged vimentin and untagged vimentin compared with those formed from untagged vimentin alone in both MCF-7 and A431 cell lines under identical experimental conditions (Figure 7). Our data suggest that N-terminally tagged vimentin is not only incapable of forming filaments, but it also reduces network stability when integrated into pre-existing filaments. This situation is comparable to mutant keratins (e.g., K14 and K5), which are reported to integrate into the existing normal keratin network in keratinocytes, and makes the network weaker, which collapses when exposed to stress such as heat [68,69,70,71]. A number of studies have used IF polypeptides (keratins, vimentin, etc.) tagged at the N-terminus, mostly with EGFP, to label the cytoskeleton for live cell imaging and fluorescence recovery after photobleaching (FRAP) assays [39,40,72,73,74]. The data obtained are extrapolated to mimic the behaviour of untagged vimentin in vivo. Our observation that a network formed from untagged and N-terminally tagged vimentin at a ratio of 1:1 is weaker than that formed from untagged vimentin alone is thought-provoking and will encourage researchers to revisit some of the observations made in the literature on IF dynamics. Other workers have used a much lower ratio of tagged and untagged vimentin (1:5) but it is not known whether the lower level of tagged vimentin would influence the network stability [39]. The disadvantage of using a lower ratio of tagged and untagged vimentin is that it will reduce the fluorescence intensity and will not be suitable for imaging experiments requiring high energy laser. It has been suggested that when using a tag, one should take into account the type of probe, linker and fusion at the N- vs. C-terminus and care should be taken not to disrupt the endogenous vimentin network by the fluorescent tag and should be preferably used in cells expressing a pre-existing vimentin network [38]. In this study we have used vimentin containing HFF-1 cells in addition to epithelial cancer lines MCF-7 and A431 devoid of vimentin, as we wanted to develop a system to study EMT induced metastasis. This study therefore provides experimental evidence to support those previously published suggestions [38].

The vital role of the head domain in filament assembly has been demonstrated by several lines of evidence including proteolytic trimming or progressive deletion from N-terminus [75,76,77], complete removal of the head domain [13], or phosphorylation of serine/threonine residues by protein kinase A and C [78], deamination of arginine residues [79] and inhibition of filament assembly by vimentin 1-96 residue fragment or by a synthetic peptide derived from the highly conserved KSSYRRMFGG motif at the N-terminus [13,31]. It has also been suggested that only the ends of the head domain are important and the middle region loops out to interact with other cellular proteins [32].

**Figure 8:**
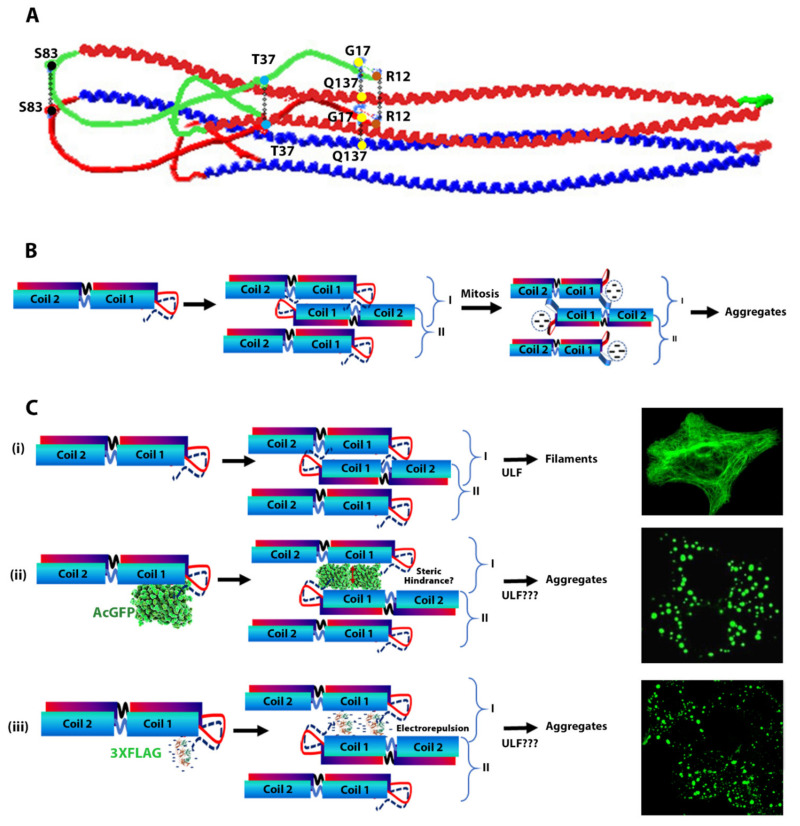
**Proposed mechanism of aggregation by N-terminally tagged vimentin.** (**A**) Vimentin tetramer structure in which the rod domains of two dimers (red and blue) associate in a staggered fashion. The head domain (shown in green for one dimer and red for the other dimer) folds back onto the rod domain causing G17 of the head domain to interact with Q137 of coil I (residues highlighted as yellow dots) (modified from [42]). (**B**) Schematic representation of the two possible ways (I and II) dimers can associate into a tetramer. The N-terminus of the vimentin head domain has a large number (8 out of 26) of serine/threonine residues, phosphorylation of which during mitosis or calyculin A treatment causes repulsion between the head domains leading to disruption of association between head–head and head–rod including G17 and Q137 interaction. This causes conformational alterations leading to collapse of the network. (**C**) **i**. Eight tetramers (both types I and II) association to form ULFs which further associate into mature filaments; **ii**. Tagging the head domain at the N-terminus with AcGFP, which is a larger tag (239 residues; 27 kDa), will therefore interfere with head–head, head–rod and rod–rod interactions (shown by a double arrow) when the head domains fold back, due to steric hindrance, and is likely to have the same effect as induced by phosphorylation of serine/threonine residues located at the N-terminus. This may affect ULF formation or render them incapable of associating into filaments; **iii**. When vimentin is tagged at the N terminus with 3 × FLAG, a much smaller tag of only 22 residues, aggregates are still formed. This can be explained by the fact that 11 out of 22 residues in 3 × FLAG are aspartic acid making the tag peptide acidic (pI = 3.97), which will create extensive repulsion disrupting not only the head–rod interactions but also inter-rod interactions affecting formation of ULF or making them unable to associate into filaments (redrawn and modified from reference [33]). The proposed mechanism of aggregation by N-terminally tagged vimentin is summarised in Supplementary Video SV1.

Tagging vimentin at the N-terminus will create an extension of the head domain and if the head has a non-helical structure as described in the literature [18,35], it is unlikely to affect rod–rod interactions which is vital for the formation of different associated intermediates and finally mature IFs. A number of studies have suggested association of the head domain with the rod domain is essential for filament assembly. In one study it has been proposed that the ends of the head domain interact and together they associate with the Val389 near the C-terminus of the rod domain of the other dimer [32,77]. Conversely, it has also been shown that the N-terminal head peptide actually associates with the start of α-helical rod domain on the same dimers forming tetramers [80]. In one study using site-directed spin labelling (SDSL) and electron paramagnetic resonance (EPR), FitzGerald and co-workers have showed that a number of residues including R12, G17, T37 and S83 in the head domain interact with the corresponding residues of the other vimentin polypeptide in a dimer [42]. They also showed that during in vitro assembly of ULF, the head domain folds back and interacts with the rod domain [33]. In these associations, a specific interaction of G17 in the head domain with Q137 in the rod domain has been identified (Figure 8A), which is essential for filament formation and stabilisation [33]. As the rod domains assemble into ULF these head domain residues (R12, G17, T37 and S83) perhaps become located in an environment of low dielectric constant (hydrophobic environment). It should be noted that about 33% of the first 26 residues in vimentin from the first methionine are either serine or threonine and phosphorylation of these residues during mitosis [81,82] or treatment of calyculin A [83,84] (a protein phosphatase inhibitor) will cause electrostatic repulsion which becomes intense in a hydrophobic environment causing separation of head domains in tetramers (Figure 8B). The electrostatic repulsion will also disrupt the interaction between residues G17 and Q137 causing destabilisation and collapse of vimentin filaments as has been reported previously in several studies [33,42,85].

The vimentin head domain tagged with relatively large and bulky AcGFP molecule at the N-terminus will also fold back during filament assembly, however, the large tag is likely to not only cause steric hindrance between rod–rod associations but also weaken the interaction between the residue G17 in the head with Q137 of the rod domain (Figure 8C(ii)). If steric hindrance between the rod domains is the only cause for filament disruption by AcGFP (239 residues, MW 27kDa) tagged at the N-terminus, it is hard to comprehend how filament disruption would be caused by a much smaller 3 × FLAG (22 residues, MW 2.4 kDa) tag. This suggests that the inter-rod associations in the ULF is extremely tight and there is not enough space to accommodate any extraneous structures. It is also interesting to note that 50% of residues in 3 × FLAG (11 out of 22) are aspartic acids which would make the tag extremely negative with isoelectric point of 3.97 [86]. This would induce electrostatic repulsion, which would be exacerbated in the medium of low dielectric constant, thereby destabilising the head–head, head–rod and rod–rod interactions, and preventing the formation of filaments (Figure 8C(iii)). The association between head and rod domains involving G17 and Q137 has only been demonstrated biochemically using SDSL and EPR in bacterially produced recombinant proteins. Our observation that a tag placed at the N-terminus will make the vimentin incapable of associating into filament strongly supports the role of head and rod association in vivo filament assembly and stabilisation.

In summary, we have provided evidence that ectopic expression of vimentin is able to form cytoplasmic IFs in two epithelial cell lines, MCF-7 and A431, which do not express endogenous vimentin. However, tagging the vimentin protein at the N-terminus with either AcGFP or 3 × FLAG tag prevented filament assembly in both cell lines, instead produced globules spreading throughout the cytoplasm and a sub-population co-aligning with β-catenin at the intercellular junctions. These globules could be converted back into filaments by untagged vimentin suggesting the aggregation was reversible. When introduced into cells containing pre-existing vimentin filaments, the tagged vimentin integrated into the filaments suggesting the tagged vimentin did not act as dominant negative. The N-terminally tagged vimentin, when integrated into untagged vimentin filaments in a 1:1 proportion, made the cytoskeleton weaker as shown by extensive disruption by diamide treatment compared to filaments formed of untagged vimentin alone. These results suggest that the presence of a tag (small or large) at the N-terminus of vimentin induces conformational alterations in the polypeptides which disrupts head–head, head–rod and rod–rod associations in ULFs preventing the formation of filaments and also affect its dynamics.

## 4. Materials and Methods

### 4.1. Cell Culture and Treatments

The MCF-7 [87], A431 [88], HFF-1 [89], MDA-MB-231 [90], SVFN3 [91], NHOF [92], SVpgC2A [93], T103C [94], HaCaT [95], HN31 [96] and TR146 [97] cell lines used in this study are listed in Table 1. These were cultured in either Dulbecco’s Modified Eagle Medium (DMEM), containing 10% (*v/v*) Foetal Calf Serum (FCS), 50 units/mL penicillin and 50 µg/mL streptomycin (pen/strep) (complete medium) or in Rheinwald Green Modified (RM^+^) growth medium (consisting of a 3:1 ratio of DMEM:F12 (Nutrient mixture Ham’s 12), with 10% FCS, pen/strep, insulin to a final concentration of 5 μg/mL, liothyronine (2 × 10^−11^ µM), transferrin 5 μg/mL, cholera toxin at 8.4 ng/mL, hydrocortisone 0.4 μg/mL, epidermal growth factor 10 ng/mL and adenine 24 μg/mL) [77]. Cells were maintained in the incubator in an atmosphere of 5% CO_2_ + 95% air at 37 °C. For the treatment of cells with diamide (Merck, cat# D3648), stock solution of 100 mM was prepared in PBS and diluted to 0.1 mM working solution in the growth medium just before use. The list of all the primary and secondary antibodies used in this study are listed in Table 2.

### 4.2. Protein Extraction and Western Blotting

Cells were cultured in 6-well plates in triplicates and when they reached 80% confluence, were washed twice with ice cold PBS and lysed using 200 µL/well Laemmli lysis buffer (2% sodium dodecyl sulphate (SDS), 20% glycerol, 125 mM Tris-HCl, pH 6.8). The colorimetric Lowry assay was performed using Bio-Rad DC™ Protein Assay kit (Cat. No. 5000116) in 96-well plate according to manufacturer’s protocol. Before loading the protein onto the gels, a final concentration of 10% (*v/v*) of 2-mercaptoethanol and 10% (*v/v*) of 0.1% (*w/v*) bromophenol blue were added to the cell lysates and heated at 95 °C in a heating block for 5 min then centrifuged at 14,000 rpm for 10 min. Supernatants were loaded onto the Tris-glycine 10% (*w/v*) continuous or 4–12% (*w/v*) acrylamide gradient gels from Invitrogen. The separated protein bands by SDS polyacrylamide gel electrophoresis (PAGE) were then transferred electrophoretically onto 0.45 µm pore size nitrocellulose membrane in a cold room. The non-specific sites on the membrane were blocked with a blocking buffer containing 5% (*w/v*) skimmed milk in TBST (15 mM Tris base, 137 mM NaCl, pH 7.5 + 0.1% Tween 20) for 60 min at RT, then the membranes were incubated overnight with the primary antibodies (Table 2) diluted in 0.5% blocking buffer at 4 °C on a shaker. The next day, the membranes were washed 3 times with TBST each for 5 min and incubated with secondary antibodies (goat anti-mouse IgG, peroxidase conjugated) or (donkey anti-rabbit IgG, peroxidase conjugated) diluted in the 0.5% blocking buffer for 1 h at RT on a shaker. ECL prime detection reagent (PRN2232, GE Healthcare, UK) and ChemiDoc (BioRad, UK) was used to visualise protein bands using *Image J* software.

### 4.3. Plasmid Constructs and cDNA Cloning

Plasmid DNA (pLPChygro, pLPCpuro-NAcGFP-GS10, pLPCpuro-NFLAG-GS10), GS10 stands for a spacer containing 10 consecutive residues of glycine and serine (GGGSGGGSGG), were digested with *Eco*RI and *Bam*HI enzymes in NEBuffer 2.1 (New England Biolabs (NEB, Hitchin, UK) reaction buffer, the mixture was incubated at 37 °C in a water bath for 2–4 h followed by dephosphorylation of ends with 1–2 µL recombinant shrimp alkaline phosphatase (NEB, Hitchin, UK) for 30 min at 37 °C. DNA fragments were separated using agarose gel electrophoresis on 1% (*w/v*) agarose in TAE (Tris-Acetic-EDTA) buffer containing 0.5 µg/mL ethidium bromide or GelRed (Sigma-Merck, Dorset UK). Full-length vimentin cDNA was amplified by PCR using Q5 DNA polymerase (NEB, Hitchin, UK) under the following conditions, initial denaturation at 98 °C for 4 min followed by 30 cycles of amplification at 98 °C for 30 s, 68 °C for 10 s, 72 °C for 2 min and final extension at 72 °C for 4 min. The forward and reverse primers used were as follows

Forward (F): 5′ AAAAAAGAATTCGCCACCATGTCCACCAGGTCCGTGTCCTCG,

Reverse (R): 5′ TTTTTTGATCCTTATTCAAGGTCATCGTGATGCTGAGAA.

The amplified cDNA was digested with *Eco*RI and *Bam*HI, purified on an agarose gel and ligated in the pLPC vectors. The ligation mixture was diluted 2–3 folds with 10 mM Tris-HCl buffer and used for transformation of NEB Stable *E. coli* (NEB, Hitchin, UK) competent cells. The constructs were named as follows:

1. pLPChygro-VIM (untagged vimentin, UTV) and pLPChygro (control vector, CV), (Figure 1B).
2. pLPCpuro-NAcGFP-GS10-VIM (AcGFP tagged at the N-terminus of full-length vimentin, AcGFP-VIM) and pLPCpuro-NAcGFP-GS10 (AcGFP) (Figure 1C)
3. pLPCpuro-NFLAG-GS10-VIM (3 × FLAG tagged at the N-terminus of full-length vimentin, FLAG-VIM) and pLPCpuro-NFLAG-GS10 (FLAG) (Figure 1D).

The original plasmid pLPC-N MYC was a gift from Titia de Lange (Addgene plasmid # 12540).

Bacterial pellet was then lysed, and DNA purified using a mini prep protocol (QIAprep Spin Miniprep kit # 28506, Qiagen Manchester, UK). Selected clones were sequenced to ensure no fortuitous change was introduced during PCR amplification. One of the confirmed clones was used to prepare maxi prep DNA (Qiagen Plasmid Maxi Kit # 12163) according to manufacturer’s protocol.

### 4.4. Recombinant Retrovirus Production

Phoenix A [98] were seeded (1.5–2 × 10^6^ cells) in 10 cm dishes coated with 10 µg/mL rat tail collagen by incubating at 37 °C for 1–2 h. The next day these cells were transfected with TransIT-LT1 transfection reagent (cat # MIR 2306; Mirus Bio) in serum free medium according to manufacturer’s protocol. Transfected cells were then selected with puromycin at a concentration of 2 µg/mL. Once the cells reached 90% confluence, the medium was changed and left for 24 h at 32 °C for virus production. Viral supernatants were harvested for 3 rounds and spun down for 1 h at 4300 rpm at 4 °C, snap frozen in liquid nitrogen and stored at −80 °C.

The construct with hygromycin selection were transfected into Phoenix E and 72 h post-transfection the supernatant was used to transduce PT67 cells [99], which were selected with 300 µg/mL hygromycin, and the viral supernatant was harvested as described above.

### 4.5. Transduction by Spinfection in Cultured Cells

MCF-7, A431 and HFF-1 cells were used for stable expression of different vimentin constructs (Figure 1B–D). Cells were seeded at a density of 50,000 cells in T25 culture flask in complete DMEM. The viral supernatant was thawed on ice (occasionally diluted 1:1 with complete DMEM) and polybrene (5 µg/mL) was added for charge neutralization for 15–20 min. Before transduction, the cells were conditioned with complete DMEM containing polybrene for 15–20 min at room temperature. The medium was then replaced with the polybrene/virus supernatant mixture. The culture flasks were spun at 1000 rpm at 32 °C for 1 h and then incubated at 32 °C for 24 h. Cells were then cultured in fresh complete DMEM at 37 °C for 2 days to ensure viral integration and protein expression. These cells were then selected with puromycin 2 µg/mL or hygromycin 300 µg/mL. All the stable cell lines made by retroviral transduction followed by drug selection used in this study are listed in Table 3.

### 4.6. Immunofluorescence Staining

Cells were cultured on collagen (10 µg/mL) coated coverslips at a density of 50,000 cells/70 µL of complete medium. After 6 to 8 h, the cells were flooded with 1 mL of the medium and washed twice with PBS and fixed with ice cold methanol and acetone (1:1) or 4% paraformaldehyde for 10 min at RT. Cells fixed with paraformaldehyde were permeabilised with 0.1% triton X-100 (*v/v*) for 20 min and washed twice with PBS for 5 min. A concentration of 10% (*v/v*) normal goat serum (NGS) diluted in washing buffer (0.01% *v/v* Tween 20 in 1 × PBS) was used as blocking buffer for 1 h to prevent non-specific binding. The cells were incubated in primary antibodies (Table 2) diluted in 10% NGS overnight at 4 °C, washed with washing buffer (0.1% (*v/v*) Tween 20 in 1x PBS + 0.02% sodium azide) 3 times for 5 min each. The secondary antibodies (goat anti-mouse Alexa Fluor 488^®^ (AF-488) IgG H+L; goat anti-mouse AF-594 IgG; goat anti-rabbit AF-488^®^ IgG H+L; goat anti-rabbit AF-594 IgG H+L) were diluted in 10% NGS and centrifuged at 15,000 rpm for 20 min, at 4 °C and incubated with the cells for 1 h at RT. The coverslips were washed 3 times for 5 min each. For nuclear staining, the cells were incubated for 2 min in 4′,6-diamidino-2-phenylindole (DAPI) (Thermo Fisher Scientific, Paisley, UK), 1:1000 dilution of 1 mM stock in H_2_O and the coverslips were mounted with ProLong^TM^ Gold Antifade Mountant and left overnight at 4 °C in the dark at RT to harden. The images were taken at BALM (Blizard Advance Light Microscopy) core facility with Leica DM4000B epi-fluorescence upright microscope (Leica Microscope, Milton Keynes, UK) or confocal microscope (Zeiss 880 laser scanning confocal microscope with Fast Airyscan and Multiphoton (inverted) system). Images were processed with *Image J* software and assembled using Microsoft PowerPoint.

### 4.7. Statistical Analysis

The experiments were performed in triplicate (technical repeats). To compare the two groups, two tailed Student’s t-tests were applied using Microsoft Excel, and *p* values below 0.05 (*p* < 0.05) were considered significant. All results were represented as the mean of 3 individual experiments (*n* = 3) with ± standard error of the mean (S.E.M).

## Figures and Tables

**Table 1:** List of cell lines used in this study.

Cell Line	Origin	Reference
TR146	Human squamous cell carcinoma	[97]
A431	Epidermoid carcinoma of the vulva	[88]
MDA-MB-231	Invasive and poorly differentiated cell line derived from breast adenocarcinoma. Derived from metastatic site: Pleural effusion	[12]
MCF-7	Epithelial cancer cell line derived from breast adenocarcinoma. Derived from metastatic site: Pleural effusion	[87]
SVpgC2a	Simian virus 40 transformed human premalignant buccal keratinocyte	[93]
SVFN3	Malignant buccal keratinocyte derived after treating SVpgC2a with nicotine and over-expression of FOXM1B	[91]
HaCaT	Spontaneously immortalized human keratinocyte line	[95]
HFF-1	HPV type 16 E6/E7 immortalised human foreskin fibroblasts	[89]
NHOF	Normal human oral keratinocytes	[92]
T103C	HPV type 16 E6/E7 immortalized oral keratinocytes	[94]
HN31	Head and neck squamous cell carcinoma line	[96]

**Table 2:** List of primary and secondary antibodies used in this study.

Antibody	Dilution	Host	Catalogue #	Supplier
Anti-vimentin, V9	IF = 1:700 WB = 1:2000	Mouse	ab8069	Abcam
Anti-cytokeratin K18	IF = 1:100 WB = 1:1000	Rabbit	ab24561	Abcam
Anti-GFP	IF = 1:500 WB = 1:10,000	Rabbit	ab183734	Abcam
Anti-GAPDH	WB = 1:2000	Rabbit	Ab9485	Abcam
Anti-FLAG	IF = 1:500 WB = 1:1000	Rabbit	F7425	Millipore UK
Mouse IgG peroxidase conjugated	WB = 1:1000	Goat	NA931V	GE Healthcare UK
Rabbit IgG peroxidase conjugated	WB = 1:1000	Donkey	NA934	GE Healthcare UK
A45-B/B3 (anti-pancytokeratin)	IF = Neat hybridoma supernatant	Mouse	In-house, neat hybridoma supernatant	In-house, neat hybridoma supernatant^40^
Anti-Integrin α6	IF = 1:100	Mouse	ab181551	Abcam
Anti-β-catenin	IF = 1:100	Mouse	610154	BD Biosciences
Anti-E cadherin	IF = 1:100	Mouse	ab1416	Abcam
Anti-vinculin	IF = 1:150	Mouse	V4505	Sigma-Aldrich
Anti-mouse Alexa Fluor^®^ 488 IgG H+L	IF = 1:1000	Goat	A-11001	Life Technologies, UK
Anti-mouse Alexa Fluor^®^ 594 IgG H+L	IF = 1:1000	Goat	A-11005	Molecular Probes, UK
Anti-rabbit Alexa Fluor^®^ 488 IgG H+L	IF = 1:1000	Goat	A-11008	Life Technologies, UK
Anti-rabbit Alexa Fluor^®^ 594 IgG H+L	IF = 1:1000	Goat	A-11012	Life Technologies, UK

**Table 3:** List of stably transduced cell lines used in this study.

Name of the Transduced Cell Lines Used in This Study	Abbreviated as
MCF-7 pLPChygro-VIM (untagged Vimentin)	MCF-7 UTV
MCF-7 pLPChygro (control vector)	MCF-7 CV
A431 pLPChygro-VIM (untagged Vimentin)	A431 UTV
A431 pLPChygro (control vector)	A431 CV
MCF-7 pLPCpuro-NAcGFP-GS10-VIM	MCF-7 AcGFP-VIM
MCF-7 pLPCpuro-NAcGFP-GS10	MCF-7 AcGFP CV
A431 pLPCpuro-NAcGFP-GS10-VIM	A431 AcGFP-VIM
A431 pLPCpuro-NAcGFP-GS10	A431 AcGFP CV
MCF-7 pLPCpuro-NFLAG-GS10-VIM	MCF-7 FLAG-VIM
MCF-7 pLPCpuro-NFLAG-GS10	MCF-7 FLAG CV
A431 pLPCpuro-NFLAG-GS10-VIM	A431 FLAG-VIM
A431 pLPCpuro-NFLAG-GS10	A431 FLAG CV
HFF-1 pLPCpuro-NAcGFP-GS10-VIM	HFF-1 AcGFP-VIM
HFF-1 pLPCpuro-NAcGFP-GS10	HFF-1 AcGFP CV
HFF-1 pLPCpuro-NFLAG-GS10-VIM	HFF-1 FLAG-VIM
HFF-1 pLPCpuro-NFLAG-GS10	HFF-1 FLAG CV

## Data Availability

Not applicable.

## Supplementary Materials

The following supporting information can be downloaded at: https://www.mdpi.com/article/10.3390/ijms23116349/s1.
