# Supplementary material for: Impact of N-Terminal Tags on De Novo Vimentin Intermediate Filament Assembly
Source: Int J Mol Sci. 2022 Jun 6;23(11):6349. doi: 10.3390/ijms23116349 (PMC9181571; doi:10.3390/ijms23116349)
Supplement: Supplementary file 1 [file ijms-23-06349-s001.zip › ijms-1689484-supplementary.pdf]

International Journal of *Molecular Sciences*

MDPI

Article

Impact of N-Terminal Tags on De Novo Vimentin Intermediate Filament Assembly

Saima Usman[1], Hebah Aldehlawi[2], Thuan Khanh Ngoc Nguyen[1], Muy-Teck Teh[1] and Ahmad Waseem[1,#]

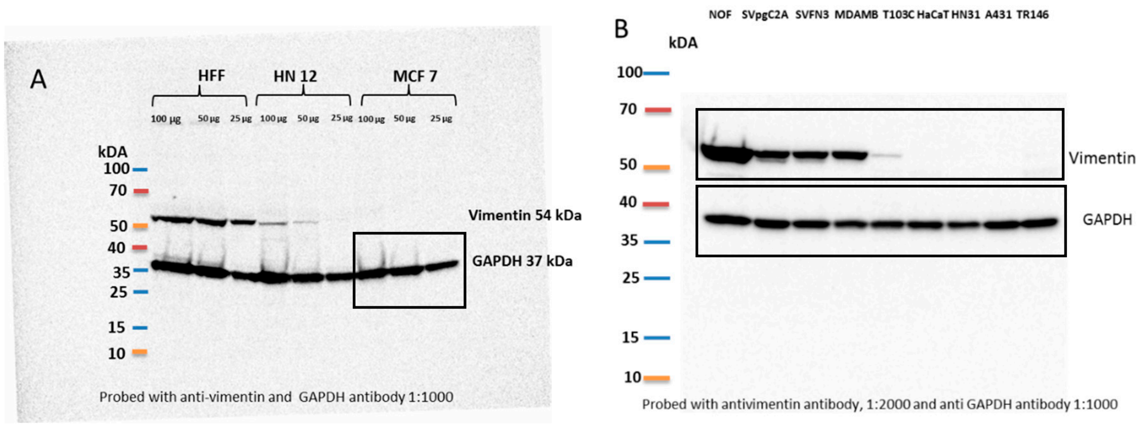

Supplementary Figure S1. (A) VIM protein expression in HFF, HN12 and MCF-7 cell lines by western blotting. 25, 50 and 100 µg of protein was loaded for each cell line. **(B)** VIM protein expression in nine different cell lines (NOF, SVpgC2A, SVFM3, MDA-MB-231, T103C, HaCaT, HN-31, A431, TR146) by western blotting. 50 µg protein was loaded for all the cell lines. GAPDH was used as the loading control in both A and B panels. Cropped areas are shown by rectangles, and these are regrouped in Figure1A.

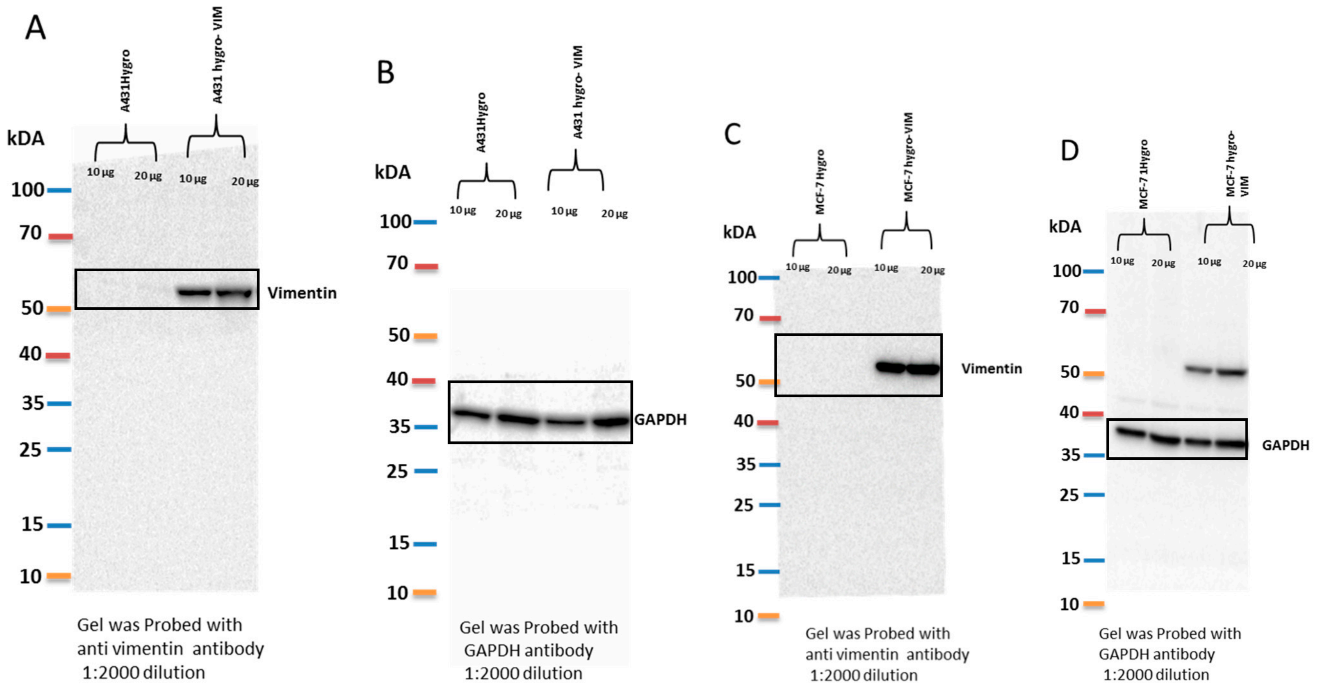

Supplementary Figure S2. Protein expression of untagged vimentin UTV (pLPChygro-VIM) and its vector control (pLPChygro) in A431 (panel A,B) and MCF-7 cells (panel C and D). Cell lines were transduced with untagged vimentin UTV (pLPChygro-VIM) and its vector control (pLPChygro). 10 and 20 µg of protein from each transduced cell line was loaded to confirm the transduction efficiency, antivimentin antibody was used for probing in 1:2000 dilution. GAPDH was used as the loading control. The cropped areas are shown by rectangle and are regrouped in Figure 2B for A431 cells (S2 panel A and B) and Figure 2A for MCF-7 cells (S2 panel C and D).

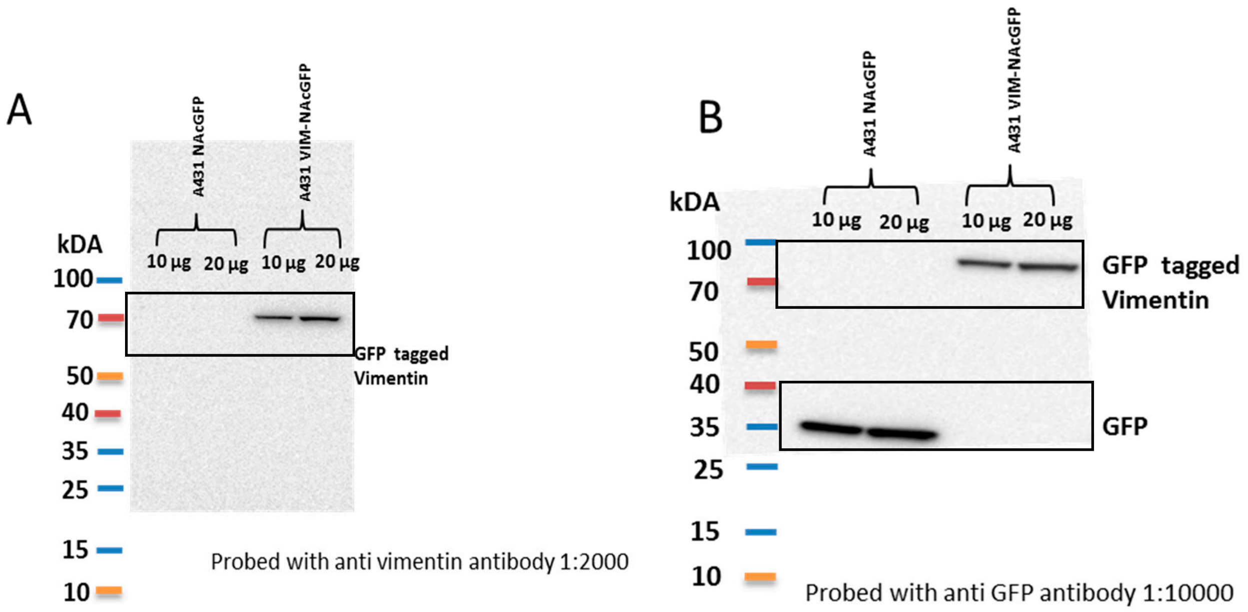

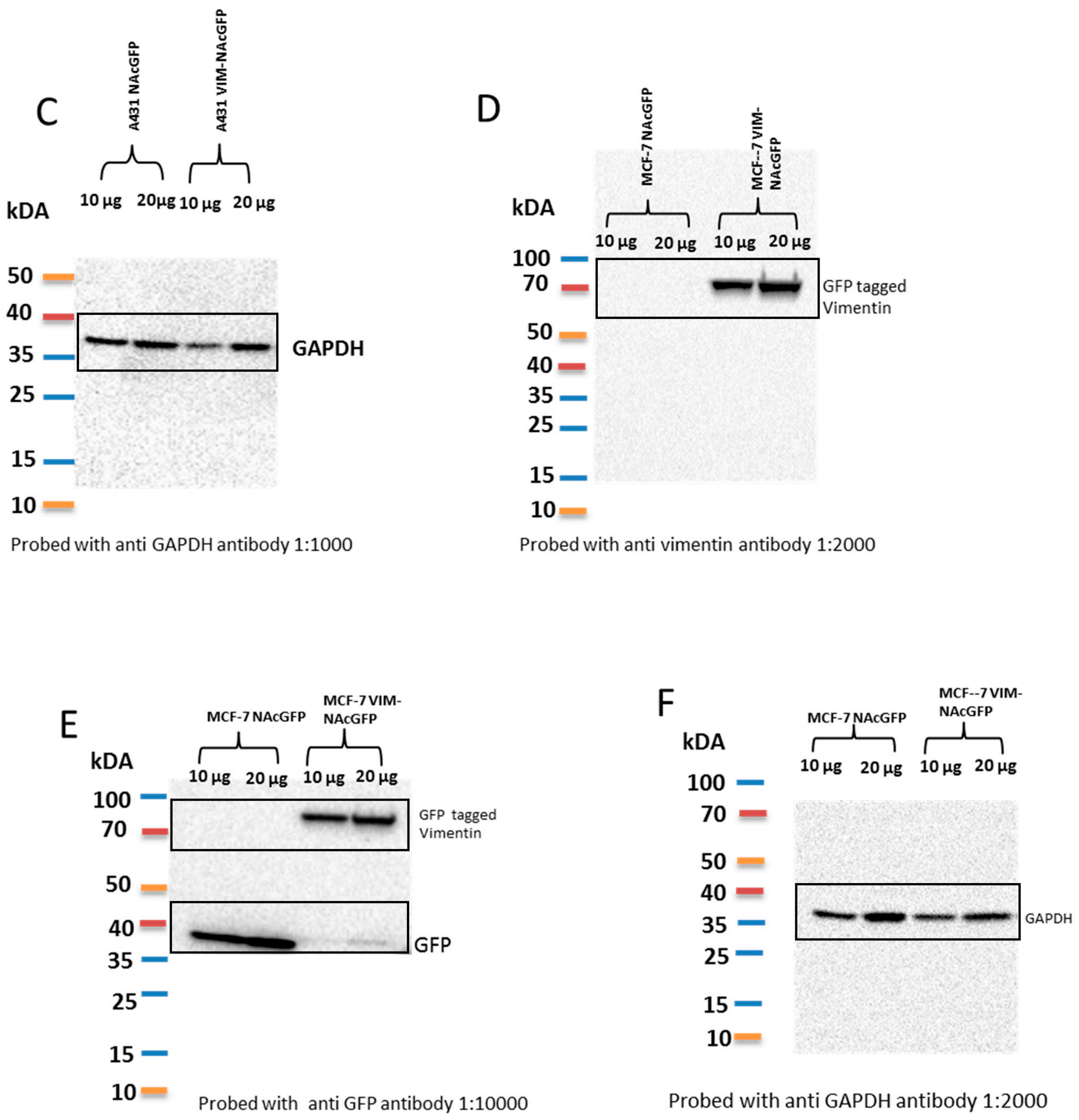

Supplementary Figure S3. Protein expression of pLPCpuro-NAcGFP-VIM and its control pLPCpuro-NAcGFP in A31 (panel A,B,C) and MCF-7 cells (panel D, E and F). Cell lines were transduced with pLPCpuro-NAcGFP-VIM and its control pLPCpuro-NAcGFP vimentin. 10 and 20 µg of protein from each transduced cell line was loaded to confirm the transduction efficiency, antivimentin and anti GFP antibodies were used for probing in 1:2000 , and 1:10000 dilutions respectively. GAPDH was used as the loading control. Cropped areas are indicated by rectangle. Panels A, B, C are regrouped in Figure 3B (A431) whereas panels D, E and F are regrouped in Figure 3A (MCF-7 cells).

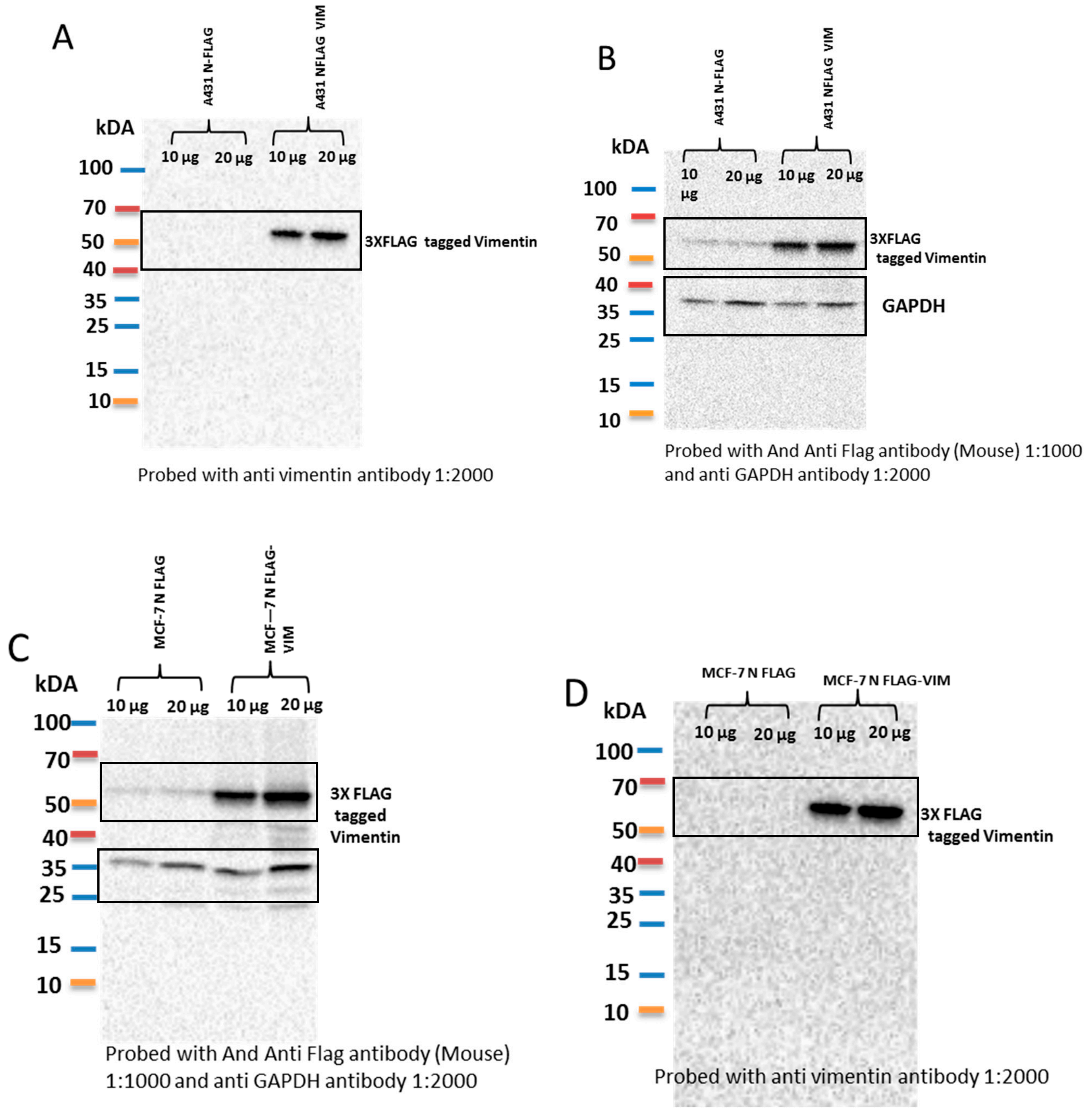

Supplementary Figure S4. Protein expression of pLPCpuro-NFLAG-VIM and its control pLPCpuro-NFLAG in A31 (panel A,B) and MCF-7 cells (panel C and D). Cell lines were transduced with **pLPCpuro-NFLAG-VIM and its control pLPCpuro-NFLAG.** 10 and 20 µg of protein from each transduced cell line was loaded to confirm the transduction efficiency, antivimentin and anti FLAG antibodies were used for probing in 1:2000 , and 1:1000 dilutions respectively. GAPDH was used as the loading control. Cropped areas are indicated by rectangle. Panels A and B are regrouped in Figure 3F (A431) whereas panels C and D are regrouped in Figure 3E (MCF-7 cells).

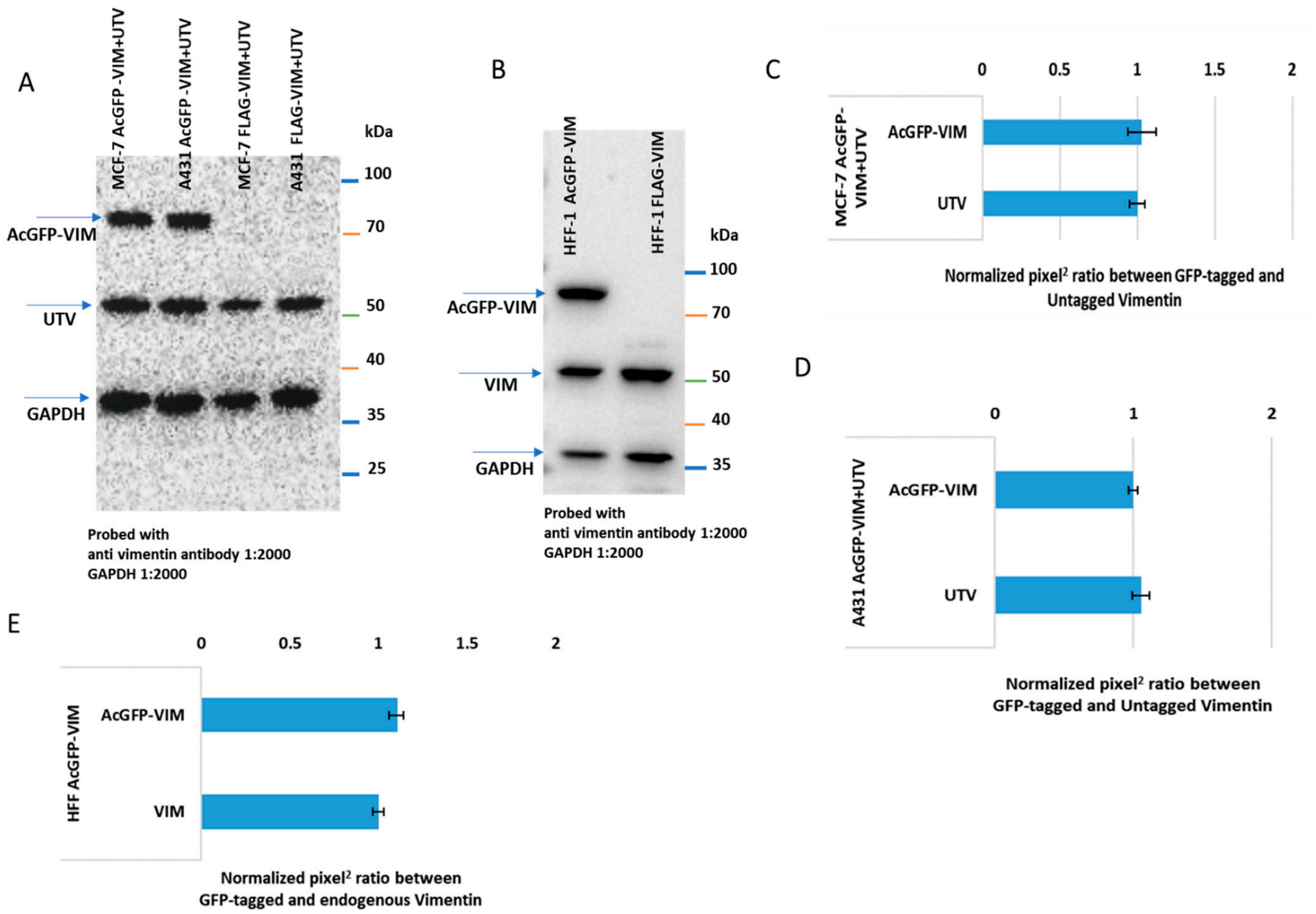

Supplementary Figure S5. Protein expression of pLPCpuro-NAcGFP-VIM + Untagged Vimentin UTV, pLPCpuro-NFLAG-VIM + Untagged Vimentin UTV in MCF-7 and A431 (panel A) and pLPCpuro-NAcGFP-VIM, pLPCpuro-NFLAG-VIM expression in HFF-1 cells (panel B). MCF-7 and A431 cell lines were transduced with pLPCpuro-NFLAG-VIM+UTV and pLPCpuro-NFLAG-VIM + UTV whereas HFF-1 cells were transduced with pLPCpuro-NAcGFP-VIM and pLPCpuro-NFLAG-VIM. Ten µg of protein from each transduced cell line was loaded to determine the ratio of untagged (endogenous in case of HFF-1) and tagged vimentin. Anti-vimentin antibody V9 was used for probing at 1:2000 dilution. GAPDH was used as the loading control. The ratio of the normalized pixel^2 intensity between AcGFP tagged and untagged vimentin was plotted for MCF-7 **(Panel C),** A431 **(Panel D)** and HFF-1 **(Panel E)** cell lines. The quantification analysis was carried out using *image J* software and showed a ration of 1:1 between tagged and untagged vimentin. 3 × FLAG tagged and untagged vimentin could not be separated on SDS PAGE due to very little (2.4 kDa) difference in their molecular weight.

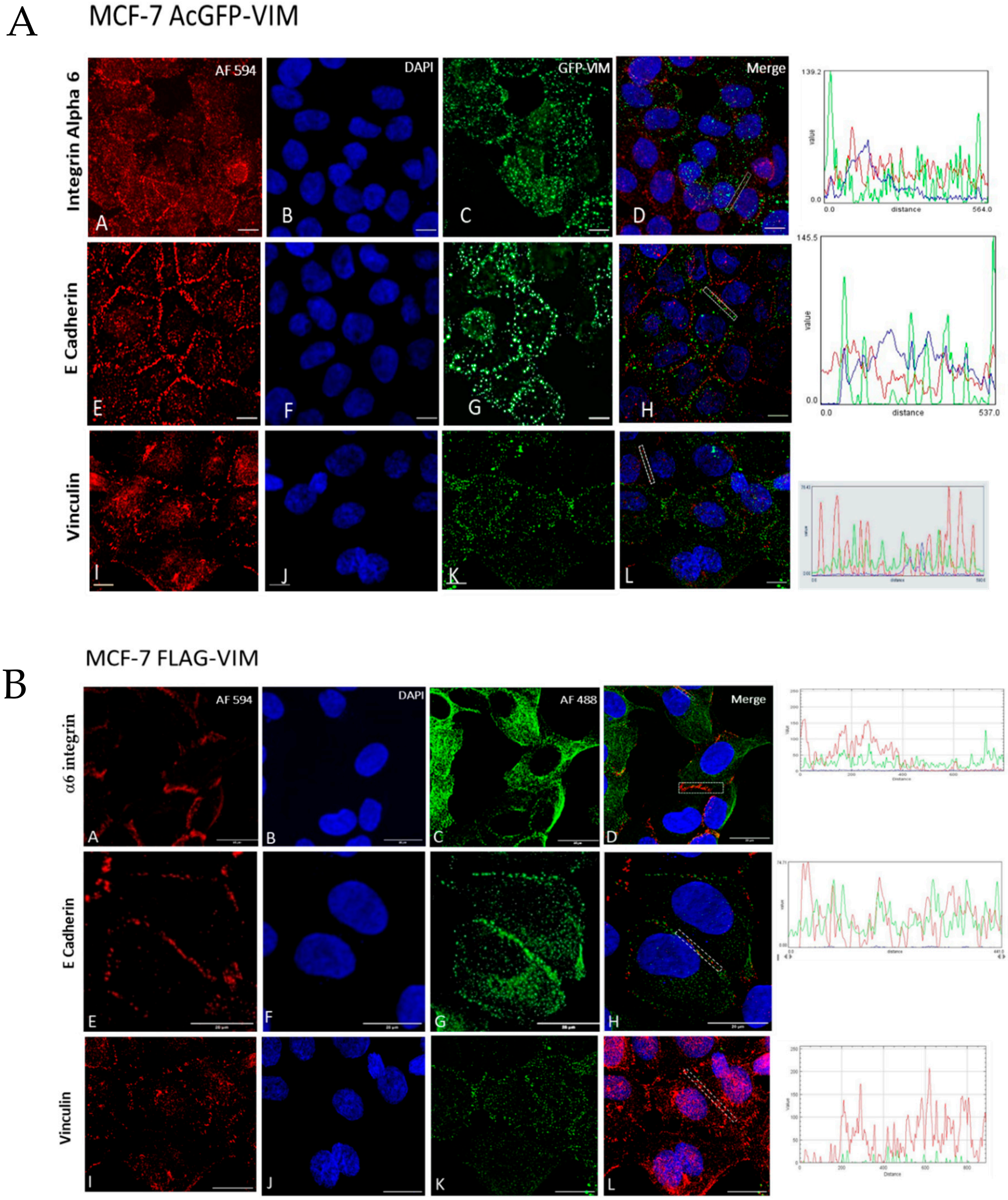
A
MCF-7 AcGFP-VIM
Integrin Alpha 6
E Cadherin
Vinculin
AF 594
DAPI
GFP-VIM
Merge
A
B
C
D
E
F
G
H
I
J
K
L
139.2
value
0.0
0.0
distance
564.0
145.5
value
0.0
0.0
distance
537.0
B
MCF-7 FLAG-VIM
α6 integrin
E Cadherin
Vinculin
AF 594
DAPI
AF 488
Merge
A
B
C
D
E
F
G
H
I
J
K
L

C

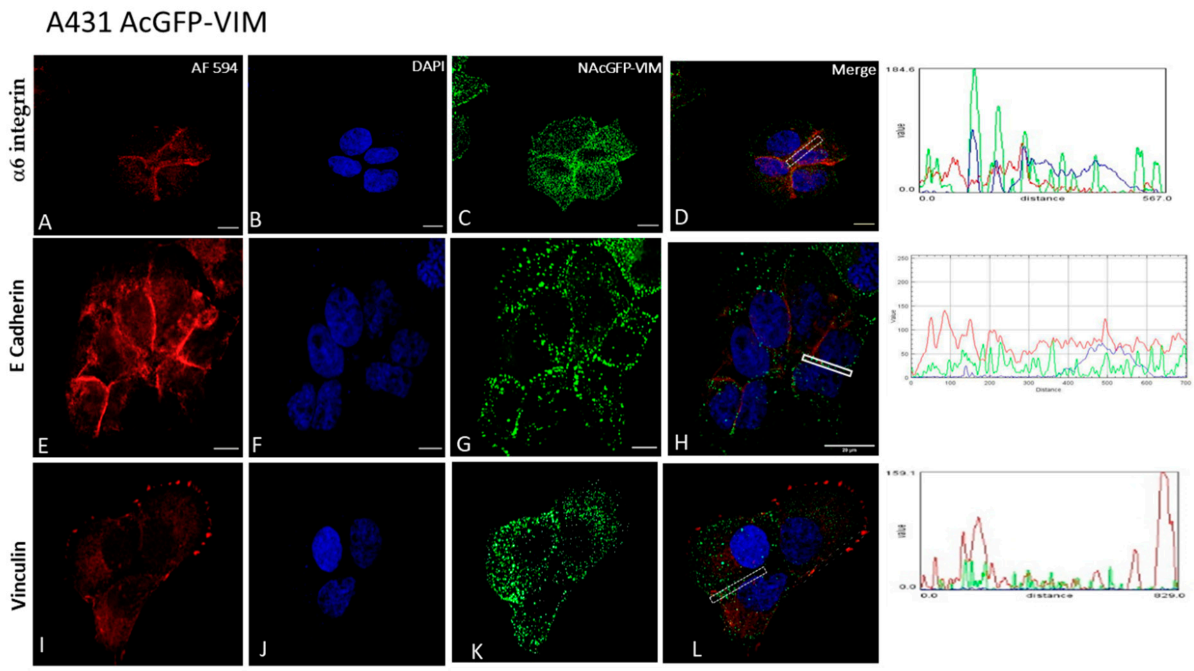

D

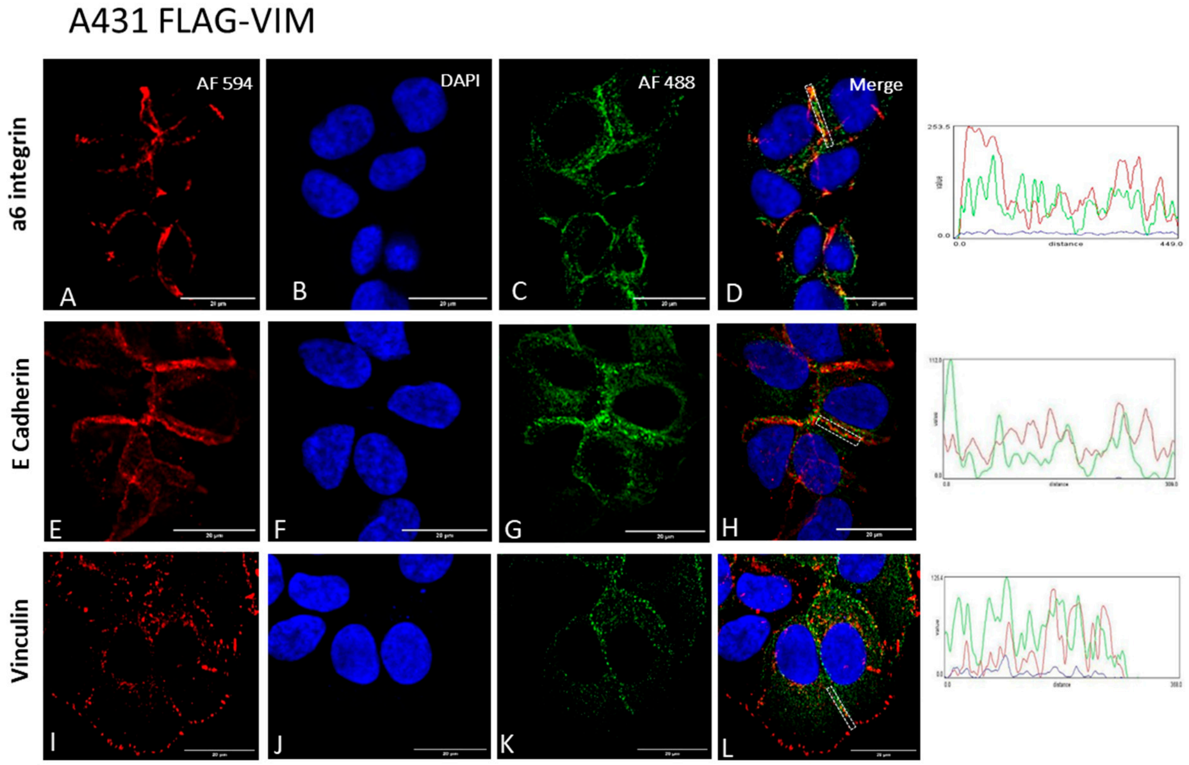

Supplementary Figure S6. Maximal single confocal sections together with RGB profiler line graph in MCF-7 AcGFP-VIM (**A**), MCF-7 FLAG-VIM (**B**), A431 AcGFP-VIM (**C**) and A431 FLAG-VIM (**D**). The cells were double immunostained with E-cadherin, vinculin or a6 integrin and anti- FLAG antibodies in case of FLAG constructs. Line graph using RGB profiler plugin shows no colocalization of red and green fluorescence signals.

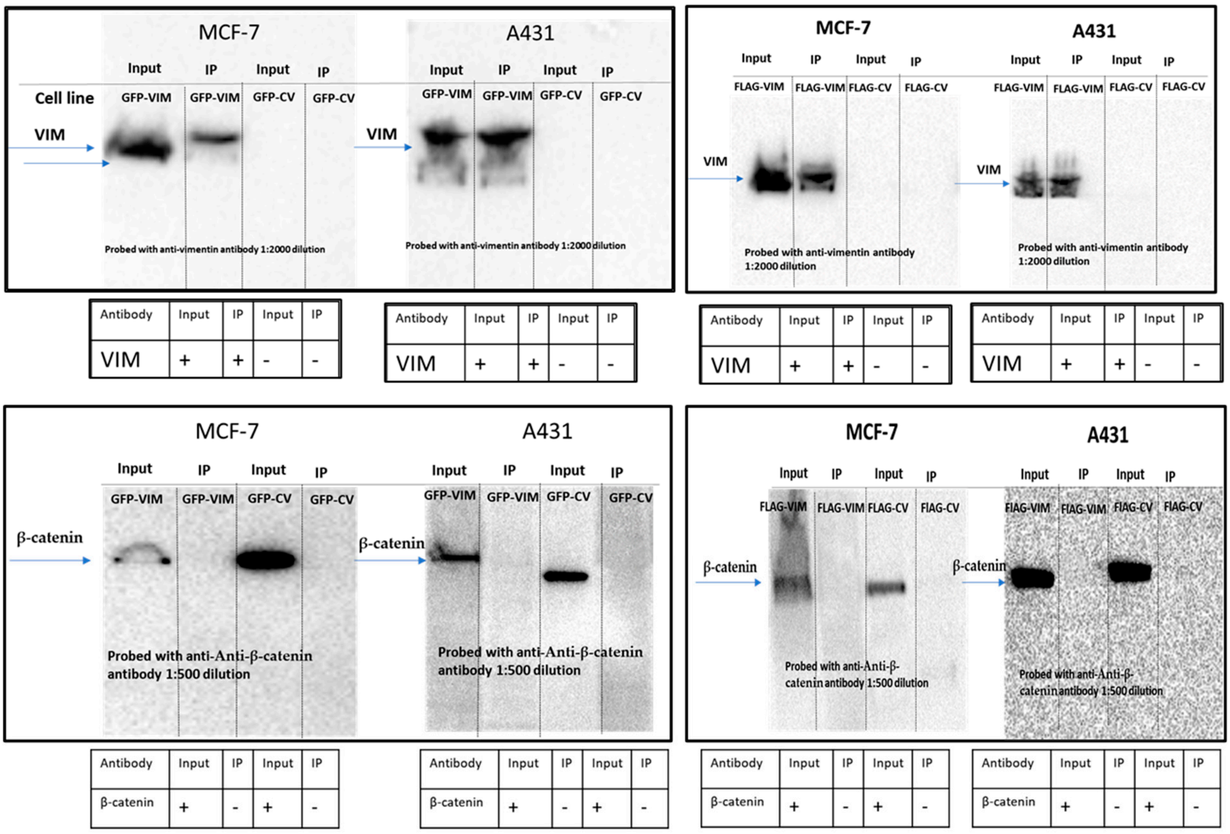

Supplementary Figure S7. Western Blot analysis of vimentin and beta catenin protein co-immunoprecipitation in MCF-7 and A431 cells expressing GFP-VIM or FLAG-VIM. Lysates from different cells lines were incubated overnight with anti-FLAG antibody M2 coated Magnetic bead or Anti-GFP antibody coated Agarose bead for FLAG and GFP constructs respectively. Immune complexes were pulled either on the magnet (For FLAG) or by centrifuging (for GFP) after boiling in Lamellae buffer containing 10 % bromophenol blue and 10% beta mercaptoethanol. 15 µl supernatant was loaded on SDS PAGE gel. The blot was probed with anti-vimentin v9 or beta catenin antibody. Input panels show the presence of vimentin and β-catenin but immunoprecipitation IP panels shows the presence of only vimentin protein.

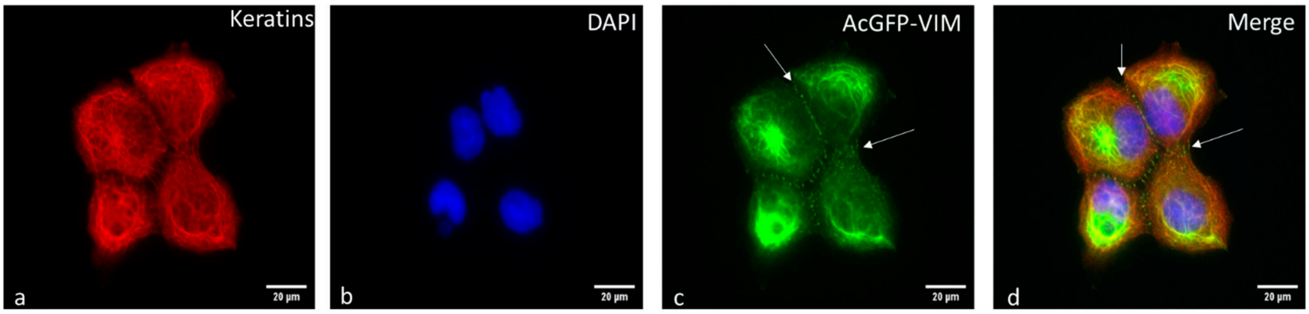

Supplementary Figure S8. Reversibility of AcGFP-tagged vimentin aggregates into filaments when untagged vimentin was transduced in A431 cells. Cells expressing AcGFP-tagged vimentin was transduced with UTV. These were stained with A45-B/B3 for keratins and AF594-labelled goat anti-mouse secondary antibody (red). Nuclei were stained with DAPI in blue and overlapping of all colours is shown as 'Merge'. Leica DM4000B Epi-fluorescence microscope was used for visualisation and DFC350 camera was used for image recording. (Scale bar = 20 µm). Note that addition of UTV can convert most of the globules into filaments, but occasionally tiny dots remain at cell-cell junction (arrows) providing the basis to speculate that untagged vimentin in the presence of N-terminally tagged vimentin globules can associate into filaments, with much higher affinity between vimentin polypeptides, so the N-terminally tagged vimentin is pulled away from the low affinity sites at the cell-cell junctions and integrated into the high affinity network formed by the untagged vimentin.
